# Clinical Characteristics of Patients With *HNF1-alpha* MODY: A Literature Review and Retrospective Chart Review

**DOI:** 10.3389/fendo.2022.900489

**Published:** 2022-06-20

**Authors:** Qinying Zhao, Li Ding, Ying Yang, Jinhong Sun, Min Wang, Xin Li, Ming Liu

**Affiliations:** ^1^ Department of Endocrinology and Metabolism, Tianjin Medical University General Hospital, Tianjin, China; ^2^ National Health Commission (NHC) Key Laboratory of Hormones and Development, Tianjin Medical University, Tianjin, China; ^3^ Tianjin Institute of Endocrinology, Tianjin, China

**Keywords:** *HNF1-alpha* MODY, diabetes, *HNF1-alpha* mutations, pancreatic islet autoantibody, diabetic complication

## Abstract

The clinical manifestation of hepatocyte nuclear factor-1-alpha (*HNF1-alpha*) maturity-onset diabetes of the young (MODY) is highly variable. This study aims to investigate the clinical characteristics of patients with *HNF1-alpha* MODY in general, by geographical regions (Asian or non-Asian), *HNF1-alpha* mutations, and islet autoantibody status. A literature review and a chart review of patients with *HNF1-alpha* MODY were performed. The means and proportions from studies were pooled using the inverse variance method for pooling, and subgroup analyses were performed. A total of 109 studies involving 1,325 patients [41.5%, 95% confidence interval (CI): 35.2, 48.1; male] were identified. The mean age of diagnosis was 20.3 years (95% CI: 18.3–22.2), and the mean glycated hemoglobin was 7.3% (95% CI: 7.2–7.5). In comparison, Asian patients exhibited significantly higher HbA1c (*p* = 0.007) and 2-h post-load C-peptide (*p* = 0.012) levels and lower levels of triglyceride (TG) (*p* < 0.001), total cholesterol (TC) (*p* < 0.001), and high-density lipoprotein cholesterol (HDL-c) (*p* < 0.001) and less often had macrovascular complications (*p* = 0.014). The age of diagnosis was oldest in patients with mutations in the transactivation domain (*p* < 0.001). The levels of 2-h post-load C-peptide (*p* < 0.001), TG (*p* = 0.007), TC (*p* = 0.017), and HDL-c (*p* = 0.001) were highest and the prevalence of diabetic neuropathy was lowest (*p* = 0.024) in patients with DNA-binding domain mutations. The fasting (*p* = 0.004) and 2-h post-load glucose (*p* = 0.003) levels and the prevalence of diabetic neuropathy (*p* = 0.010) were higher among patients with positive islet autoantibodies. The study demonstrated that the clinical manifestations of *HNF1-alpha* MODY differed by geographical regions, *HNF1-alpha* mutations, and islet autoantibody status.

## Introduction

Maturity-onset diabetes of the young (MODY) is a monogenic diabetes syndrome, characterized by onset before 25–35 years of age, autosomal dominant inheritance, negative islet autoimmunity, and lack of the typical features of type 2 diabetes (T2DM). Mutation in the hepatocyte nuclear factor-1-alpha (*HNF1-alpha*) gene is one of the most common causes of MODY (1–5). The *HNF1-alpha* MODY phenotype is characterized by early-onset diabetes and progressive β-cell dysfunction with defective insulin secretion (6). *HNF1-alpha* MODY is caused by the mutation of the *HNF1-alpha* gene, consisting of dimerization domain, DNA-binding domain, and transactivation domain, which regulates multiple genes involved in glucose metabolism in the pancreas, kidney, and liver (7, 8). Though the clinical manifestations of *HNF1-alpha* MODY overlap with type 1 diabetes mellitus (T1DM) and T2DM, patients with *HNF1-alpha* MODY are generally more sensitive to sulfonylureas (9, 10). Thus, the accurate identification of *HNF1-alpha* MODY is of utmost importance for the optimal management and prevention of diabetes-associated micro- and macrovascular complications, which are not infrequent in *HNF1-alpha* MODY.

About 1–5% diabetes and 0.83–6.5% of diabetic children and adolescents were identified as MODY (2, 11–16). The prevalence of *HNF1-alpha* MODY varies among nations and healthcare systems. In a study involving 101 families (95% Caucasian in the United Kingdom), Frayling *et al.* found that 63% of patients with MODY fit the *HNF1-alpha* MODY criteria (17). In a retrospective observational study involving 565 children and adolescents with newly diagnosed diabetes in Southern Italy, *HNF1-alpha* MODY accounted for 13.5% of MODY cases (2). The detection rate of *HNF1-alpha* MODY was 13.9% in Japanese (18) and 15.79% in Chinese pedigree MODY genetic screening studies (19). Previous studies showed that genetic modifiers and *in utero* exposure to hyperglycemia led to a variability in the clinical presentation of *HNF1-alpha* MODY (20, 21). It is unclear if the genetic and environmental factors of different geographic regions may result in different phenotypes of patients with *HNF1-alpha* MODY.

Severe hyperglycemia usually occurs after puberty in patients with *HNF1-alpha* MODY, which may lead to the misdiagnosis of T1DM. Genetic testing that is required for the diagnosis of *HNF1-alpha* MODY is usually sought only in individuals negative for islet autoantibodies, including glutamic acid decarboxylase antibody (GAD), protein tyrosine phosphatase antibody (IA2), and islet cell antibody (ICA) (22), and individuals originally considered as T1DM but who were negative for islet autoantibodies (12). However, some studies reported that a proportion of patients with *HNF1-alpha* MODY were positive for islet autoantibodies (15, 23–30). There is currently a lack of research on whether the clinical phenotype of *HNF1-alpha* MODY differs by geographic regions, *HNF1-alpha* mutations, and islet autoantibody status. In this study, we analyzed the clinical characteristics of patients with *HNF1-alpha* MODY in general and by geographical regions, *HNF1-alpha* mutations, and islet autoantibody status through literature and chart review.

## Materials and Methods

### Study Selection

A literature search was conducted through Pubmed, Web of Science, Embase, Wanfang, and the China National Knowledge Infrastructure Databases from inception of the database to December 2021 using medical subject headings or Emtree thesaurus as well the following key terms: “Maturity-Onset Diabetes of the Young, Type 3”, “MODY3”, “MODY, Type 3”, “hepatic nuclear factor 1 alpha”, “*HNF1A*”, “*HNF1-alpha*”, “nuclear protein LF-B1”, “LF-B1 transcription factor, human”, and “HNF1 homeobox A protein, human”. A detailed search strategy for database is listed in the **Supplementary Material**. The search results were imported into endnote software where duplicated results were identified based on the title, journal, publication year, and authors and were removed automatically. The remaining references were screened through the title and abstract for relevance to this study. Studies dedicated to animal or *in vitro* experiments were excluded. The relevant studies identified, *i*.*e*., studies reporting the clinical characteristics of patients with *HNF1-alpha* MODY, were subjected to full-text review for eligibility. The eligible studies met the following criteria: (1) the diagnosis of *HNF1-alpha* MODY was confirmed by genetic testing and (2) individual-level or aggregate data were reported for at least one of the following: fasting plasma glucose (FPG), 2-h post-load glucose (2h PG) in an oral glucose tolerance test, or glycated hemoglobin (HbA1c).

### Data Extraction

Clinical data, including the geographical region, gender, age of diagnosis, body mass index (BMI), familial history, HbA1c, FPG, 2h PG, fasting and 2-h post-load C-peptide, triglyceride (TG), total cholesterol (TC), high-density lipoprotein cholesterol (HDL-c), low-density lipoprotein cholesterol (LDL-c), islet autoantibody status, diabetic complications, and anti-diabetic therapies, were abstracted. Besides this, information regarding amino acid substitution, position, and type of mutations in the *HNF1-alpha* gene were abstracted. Literature search, study selection, and data extraction were conducted by two independent reviewers, and disagreement was resolved by consensus with a third reviewer.

### Statistical Analysis

Statistical analysis was conducted using R, version 3.5.3 (http://www.r-project.org/). The means and percentages from case reports were calculated and pooled with aggregated data from cohort studies and case series using the inverse variance method for pooling in random effect models with the metamean and metaprop function in the meta package in R. The results were presented with means, proportions, and respective 95% confidence intervals. A comparison among subgroups was performed. *P*-value less than 0.05 was statistically significant.

## Results

**Figure 1:**
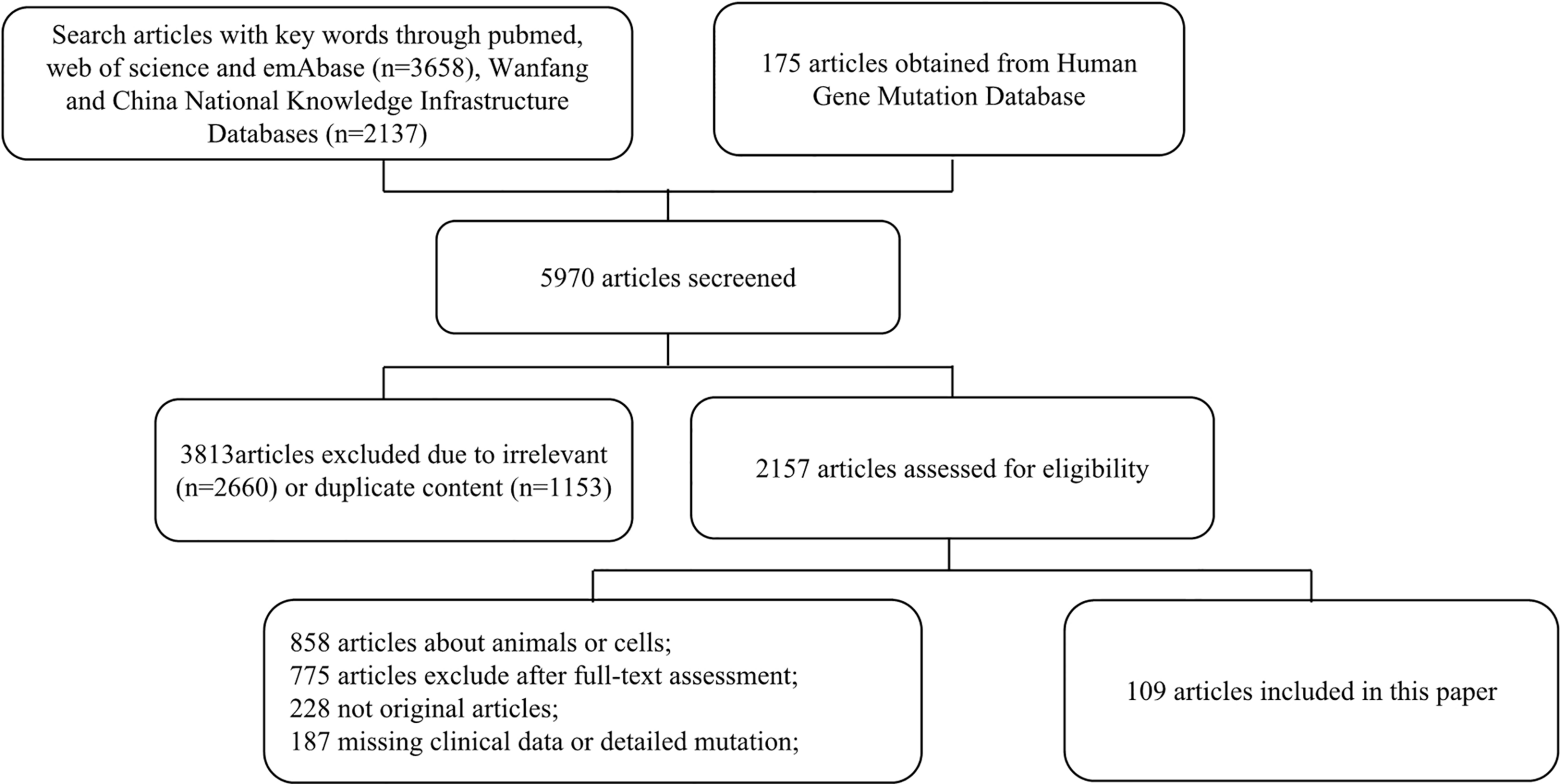
Flow chart.

Among 5,970 publications identified, 1,153 articles were excluded due to duplication, and 2,660 articles were excluded due to being irrelevant after screening through the title and the abstract. After a full-text review, 109 studies involving 1,325 patients were identified (**Figure 1**). Detailed information and citations of these included studies are provided in the supplementary material (**Supplementary Table S1**).

### Clinical Manifestations of *HNF1-alpha* MODY in General

A total of 1,325 patients [41.5%, 95% confidence interval (CI): 35.2, 48.1; male] with *HNF1-alpha* MODY were included in our study. The mean values of the clinical data were as follows: age, 32.6 years (95% CI: 28.5–36.7); age of diagnosis, 20.3 years (95% CI: 18.3–22.2); BMI, 23.1 kg/m^2^ (95% CI: 22.3–23.9); HbA1c, 7.3% (95% CI: 7.2–7.5); and FPG, 8.1 mmol/L (95% CI: 7.6–8.5). Among the patients identified, 89.8% (95% CI: 54.1–98.5) had a family history of diabetes, 47.6% (95% CI: 30.6–65.2) had microvascular complications, 21.5% (95% CI: 14.5–30.8) had diabetic retinopathy, 16.6% (95% CI: 10.3–25.5) had diabetic kidney disease, 11.8% (95% CI: 6.2–21.2) had diabetic neuropathy, 11.1% (95% CI: 7.3–16.6) had macrovascular complications, 17.0% (95% CI: 13.2–21.6) received lifestyle management, 40.3% (95% CI: 32.4–48.6) were prescribed with oral hypoglycemic drugs, 35.5% (95% CI: 31.3–40.0) were prescribed with insulin, and 9.5% (95% CI: 5.4–16.2) were prescribed with oral hypoglycemic drugs plus insulin.

### Clinical Manifestations of *HNF1-alpha* MODY by Geographical Regions

Among 109 studies identified, 37 studies involving 183 patients were from Asia, while 72 studies including 1,142 patients were from geographic regions other than Asia. Compared to non-Asian patients, BMI [20.8 kg/m^2^ (95% CI: 20.3–21.3) vs. 24.1 kg/m^2^ (95% CI: 23.6–24.6), *p* < 0.001], TG [1.14 mmol/L (95% CI: 0.81–1.48) vs. 2.09 mmol/L (95% CI: 1.69–2.49), *p* < 0.001], TC [4.70 mmol/L (95% CI: 4.46–4.94) vs. 5.76 mmol/L (95% CI: 5.23–6.29), *p* < 0.001], and HDL-c [1.37 mmol/L (95% CI: 1.29–1.45) vs. 1.75 mmol/L (95% CI: 1.59–1.92), *p* < 0.001] were lower in Asian patients. Their HbA1c [7.9% (95% CI: 7.5–8.4) vs. 7.3% (95% CI: 7.1–7.4), *p* = 0.007] and 2-h post-load C-peptide levels were higher [2.16 ng/ml (95% CI: 1.61–2.72) vs. 1.44 ng/ml (95% CI: 1.34–1.54), *p* = 0.012]. They less often had macrovascular complications [2.7% (95% CI: 0.7–10.0) vs. 14.3% (95% CI: 10.7–18.9), *p* = 0.014], while age, age of diagnosis, diabetes duration, gender, family history, the prevalence of microvascular complications, diabetic retinopathy, diabetic kidney disease, diabetic neuropathy, and anti-diabetic therapies did not differ by geographical regions (**Table 1**).

**Table 1:** Characteristics of patients with *HNF1-alpha* MODY by geographical regions.

Subject	Total (*n* = 1,325)	Asian (*n* = 183)	Non-Asian (*n* = 1,142)	*P*-value
Age (years)	32.6 (28.5, 36.7)	29.4 (24.9, 33.9)	33.5 (28.6, 38.5)	0.229
Age of diagnosis (years)	20.3 (18.3, 22.2)	18.0 (14.8, 21.3)	20.9 (18.8, 23.0)	0.153
Duration (years)	13.1 (10.5, 15.6)	13.6 (9.3, 18.0)	13.0 (10.0, 16.0)	0.809
BMI (kg/m^2^)	23.1 (22.3, 23.9)	20.8 (20.2, 21.3)	24.1 (23.6, 24.6)	<0.001*
HbA1c (%)	7.3 (7.2, 7.5)	7.9 (7.5, 8.4)	7.3 (7.1, 7.4)	0.007*
FPG (mmol/L)	8.1 (7.6, 8.5)	7.9 (7.0, 8.8)	8.1 (7.5, 8.7)	0.618
2h PG (mmol/L)	15.6 (14.8, 16.5)	16.1 (14.5, 17.8)	15.3 (14.4, 16.3)	0.432
Fasting C-peptide (ng/ml)	1.15 (0.84, 1.46)	0.96 (0.46, 1.46)	1.25 (0.86, 1.65)	0.360
2-hour post-load C-peptide (ng/ml)	2.00 (1.40, 2.59)	2.16 (1.61, 2.72)	1.44 (1.34, 1.54)	0.012*
TG (mmol/L)	1.79 (1.49, 2.09)	1.14 (0.81, 1.48)	2.09 (1.69, 2.49)	<0.001*
TC (mmol/L)	5.46 (5.04, 5.88)	4.70 (4.46, 4.94)	5.76 (5.23, 6.29)	<0.001*
HDL-c (mmol/L)	1.66 (1.52, 1.79)	1.37 (1.29, 1.45)	1.75 (1.59, 1.92)	<0.001*
LDL-c (mmol/L)	2.83 (2.60, 3.06)	2.63 (2.40, 2.87)	2.94 (2.58, 3.31)	0.161
Male (%)	41.5 (35.2, 48.1)	43.0 (33.5, 53.1)	41.0 (33.7, 48.7)	0.751
Family history (%)	89.8 (54.1, 98.5)	92.6 (22.2, 99.8)	87.4 (41.5, 98.5)	0.794
Microvascular complications (%)	47.6 (30.6, 65.2)	31.7 (5.1, 79.9)	51.6 (33.3, 69.5)	0.474
Diabetic retinopathy (%)	21.5 (14.5, 30.8)	22.2 (6.0, 56.2)	20.7 (13.9, 29.7)	0.913
Diabetic kidney disease (%)	16.6 (10.3, 25.5)	10.7 (2.8, 32.9)	18.4 (11.0, 29.1)	0.417
Diabetic neuropathy (%)	11.8 (6.2, 21.2)	6.8 (0.7, 43.5)	13.1 (7.2, 22.7)	0.559
Macrovascular complications (%)	11.1 (7.3, 16.6)	2.7 (0.7, 10.0)	14.3 (10.7, 18.9)	0.014*
Lifestyles (%)	17.0 (13.2, 21.6)	15.5 (10.6, 22.3)	16.8 (12.3, 22.5)	0.759
OHA (%)	40.3 (32.4, 48.6)	41.9 (34.2, 50.0)	40.5 (30.8, 51.0)	0.829
INS (%)	35.5 (31.3, 40.0)	35.8 (28.5, 43.8)	35.1 (30.3, 40.3)	0.882
OHA + INS (%)	9.5 (5.4, 16.2)	8.1 (3.7, 16.9)	9.9 (4.7, 19.6)	0.717

BMI, body mass index; HbA1c, glycated hemoglobin; FPG, fasting plasma glucose; 2h PG, 2-h post-load glucose; TG, triglyceride; TC, total cholesterol; HDL-c, high-density lipoprotein cholesterol; LDL-c, low-density lipoprotein cholesterol; OHA, oral hypoglycemic drugs; INS, insulin; *p < 0.05.

### Clinical Manifestations of *HNF1-alpha* MODY by Mutations

**Figure 2:**
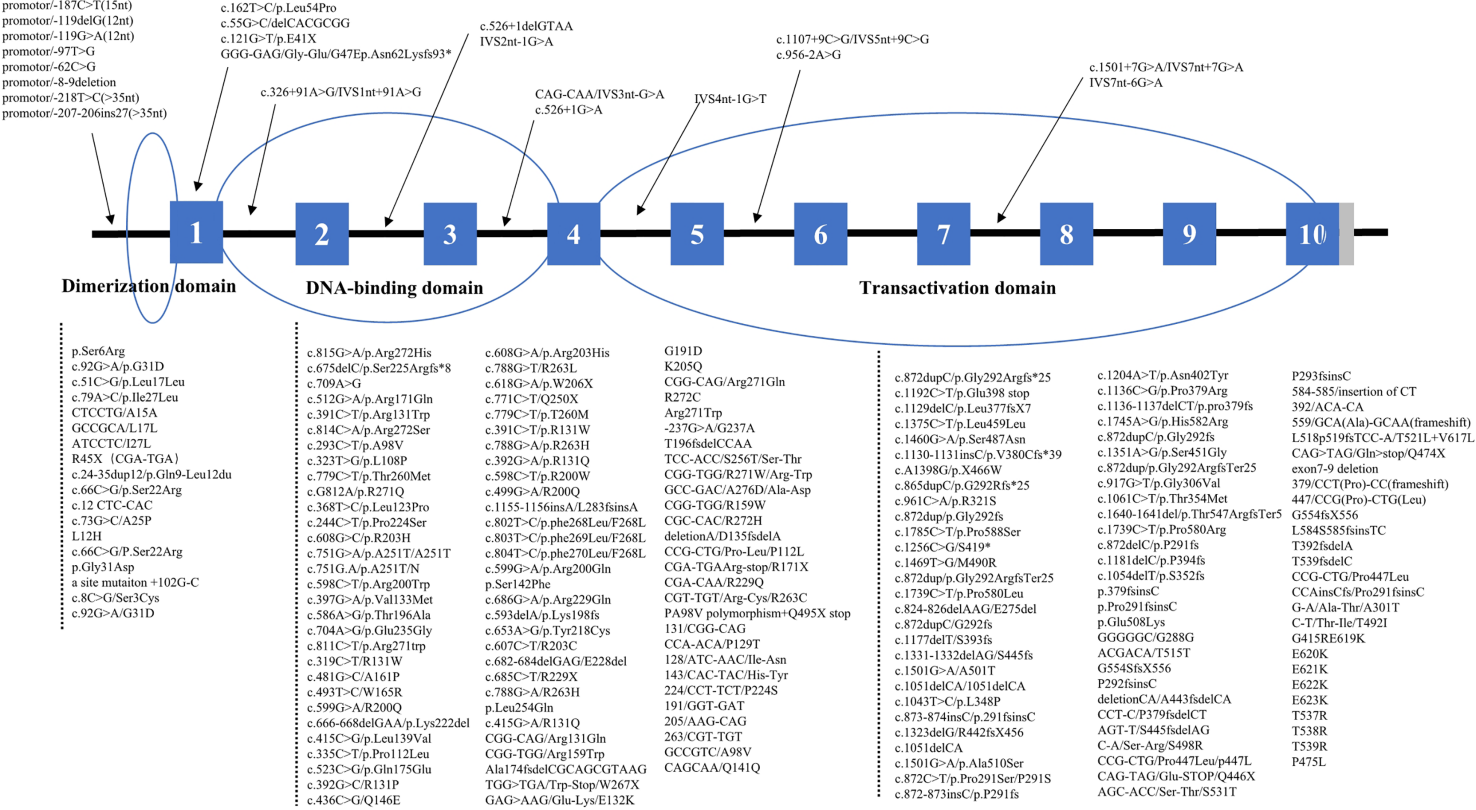
Distribution of *HNF1-alpha* mutations. *means nucleotide number and indicates translation stop codon.

Among those reporting the site and type of mutations of the *HNF1-alpha* gene, 432 patients had mutations in coding regions, consisting of 15 (3.5%) in the dimerization domain, 179 (41.4%) in the DNA-binding domain, and 238 (55.1%) in the transactivation domain (**Figure 2**). Age of diagnosis was oldest in patients with mutations in the transactivation domain [26.0 years (95% CI: 21.7–30.3) vs. 18.9 years (95% CI: 11.5–26.3) for the dimerization domain and 20.5 years (95% CI: 17.3–23.7) for the DNA-binding domain, *p* < 0.001]. The levels of 2-h post-load C-peptide [4.22 ng/ml (95% CI: 2.17–6.26) vs. 2.71 ng/ml for the dimerization domain and 1.92 ng/ml (95% CI: 1.17–2.67) for the transactivation domain, *p* < 0.001], TG [1.82 mmol/L (95% CI: 0.27–3.36) vs. 1.70 mmol/L for the dimerization domain and 1.23 mmol/L (95% CI: 0.81–1.64) for the transactivation domain, *p* = 0.007], TC [5.39 mmol/L (95% CI: 2.07–8.71) *vs*. 3.96 mmol/L for the dimerization domain and 4.54 mmol/L (95% CI: 3.59–5.49) for the transactivation domain, *p* = 0.017], and HDL-c [1.59 mmol/L (95% CI: 0.64–2.54) vs. 0.72 mmol/L for the dimerization domain and 1.12 mmol/L (95% CI: 0.89–1.35) for the transactivation domain, *p* = 0.001] were highest, while the prevalence of diabetic neuropathy was lowest [10.9% (95% CI: 5.0–22.2) vs. 33.3% (95% CI: 8.4–73.2) for the dimerization domain and 37.0% (95% CI: 21.2–56.2) for the transactivation domain, *p* = 0.024] in patients with DNA-binding domain mutations. BMI, HbA1c, FPG, 2h PG, fasting C-peptide, LDL-c, family history, gender, diabetic retinopathy, diabetic kidney disease, macrovascular complications and anti-diabetic therapies did not differ significantly (**Table 2**). The mutation domain did not differ significantly between Asian and non-Asian patients (**Table 3**).

**Table 2:** Characteristics of patients with *HNF1-alpha* MODY by genetic domains.

Subject	Total (*n* = 432)	Dimerization domain (*n* = 15)	DNA-binding domain (*n* = 179)	Transactivation domain (*n* = 238)	*P*-value
Age of diagnosis (years)	19.7 (17.6, 21.8)	18.9 (11.5, 26.3)	20.5 (17.3, 23.7)	26.0 (21.7, 30.3)	<0.001*
BMI (kg/m^2^)	22.8 (22.1, 23.6)	22.8 (20.9, 24.7)	23.6 (22.2, 25.1)	22.1 (20.8, 23.5)	0.227
HbA1c (%)	8.0 (7.6, 8.4)	8.0 (6.5, 9.5)	8.2 (7.4, 9.0)	7.8 (7.2, 8.5)	0.641
FPG (mmol/L)	9.3 (8.0, 10.5)	12.4 (4.6, 20.3)	9.1 (7.2, 11.0)	8.7 (7.6, 9.8)	0.126
2h PG (mmol/L)	15.9 (14.9, 16.9)	16.8 (8.6, 25.1)	16.1 (12.8, 19.4)	15.5 (13.0, 18.0)	0.751
Fasting C-peptide (ng/ml)	1.14 (1.03, 1.25)	1.28 (0.71, 1.85)	1.13 (0.87, 1.40)	1.04 (0.73, 1.35)	0.370
2-h post-load C-peptide (ng/ml)	3.06 (0.81, 5.32)	2.71	4.22 (2.17, 6.26)	1.92 (1.17, 2.67)	<0.001*
TG (mmol/L)	1.51 (0.93, 2.08)	1.70	1.82 (0.27, 3.36)	1.23 (0.81, 1.64)	0.007*
TC (mmol/L)	4.97 (4.13, 5.81)	3.96	5.39 (2.07, 8.71)	4.54 (3.59, 5.49)	0.017*
HDL-c (mmol/L)	1.34 (0.88, 1.81)	0.72	1.59 (0.64, 2.54)	1.12 (0.89, 1.35)	0.001*
LDL-c (mmol/L)	3.01 (2.82, 3.21)	3.02 (1.81, 4.24)	2.89 (1.18, 4.24)	3.19 (2.24, 4.14)	0.662
Male (%)	40.0 (29.1, 52.0)	46.2 (22.4, 71.8)	47.8 (37.7, 58.1)	28.3 (17.8, 41.8)	0.072
Family history (%)	83.9 (75.2, 90.0)	92.9 (63.0, 99.0)	80.4 (55.3, 93.2)	83.0 (75.7, 88.4)	0.618
Microvascular complications (%)	36.6 (26.2, 48.3)	50.0 (16.8, 83.2)	28.9 (17.6, 43.6)	41.5 (24.8, 60.4)	0.406
Diabetic retinopathy (%)	26.1 (16.8, 38.3)	33.3 (8.4, 73.2)	22.0 (11.8, 37.1)	28.6 (12.8, 52.2)	0.760
Diabetic kidney disease (%)	14.0 (7.5, 24.8)	16.7 (2.3, 63.1)	23.5 (12.2, 40.5)	8.4 (3.0, 21.3)	0.209
Diabetic neuropathy (%)	19.8 (9.9, 35.6)	33.3 (8.4, 73.2)	10.9 (5.0, 22.2)	37.0 (21.2, 56.2)	0.024*
Macrovascular complications (%)	5.3 (1.7, 15.1)	0 (0, 100)	8.3 (2.1, 27.9)	3.7 (0.5, 22.1)	0.792
Lifestyles (%)	19.4 (14.7, 25.2)	16.7 (4.2, 47.7)	18.8 (13.4, 25.8)	20.8 (13.7, 30.3)	0.903
OHA (%)	40.4 (29.6, 52.2)	66.7 (37.6, 86.9)	32.6 (15.2, 56.7)	42.6 (34.4, 51.3)	0.192
INS (%)	28.5 (24.2, 33.3)	16.7 (4.2, 47.7)	27.9 (21.4, 35.5)	29.7 (23.9, 36.2)	0.620
OHA + INS (%)	4.2 (1.9, 9.0)	0 (0, 100)	5.9 (2.7, 12.5)	3.5 (0.9, 12.6)	0.799

BMI, body mass index; HbA1c, glycated hemoglobin; FPG, fasting plasma glucose; 2h PG, 2-h post-load glucose; TG, triglyceride; TC, total cholesterol; HDL-c, high-density lipoprotein cholesterol; LDL-c, low-density lipoprotein cholesterol; OHA, oral hypoglycemic drugs; INS, insulin; *p < 0.05.

**Table 3:** Differences in domain mutations between Asian patients and non-Asian patients.

Subject	Total (*n* = 1,325)	Asian (*n* = 183)	Non-Asian (*n* = 1,142)	*P*-value
Dimerization domain (%)	6.4 (3.9, 10.4)	3.8 (1.2, 11.0)	7.8 (4.5, 13.2)	0.241
DNA-binding domain (%)	33.0 (17.2, 53.9)	48.8 (38.0, 59.6)	29.2 (12.9, 53.6)	0.144
Transactivation domain (%)	59.9 (40.7, 76.4)	47.5 (36.8, 58.4)	63.0 (40.0, 81.4)	0.231

In our study, the *HNF1-alpha* mutations included missense mutations (*n* = 227, 46.1%), frameshift mutations (*n* = 198, 40.2%), nonsense mutations (*n* = 30, 6.1%), synonym mutations (*n* = 2, 0.4%), and non-coding mutations (*n* = 35, 7.1%). Patients with non-coding mutations had the highest levels of HbA1c [9.5% (95% CI: 8.0–11.0), *p* < 0.001] and 2h PG [20.7 mmol/L (95% CI: 19.7–21.8), *p* < 0.001] and the lowest BMI [20.5 kg/m^2^ (95% CI: 19.5–21.6), *p* < 0.001] and fasting C-peptide [0.43 ng/ml (95% CI: 0.33–0.53), *p* < 0.001], and they most often had diabetic kidney disease [80.0% (95% CI: 30.9–97.3), *p* = 0.046]. In terms of lipid profiles, due to the incomplete data of patients with nonsense, synonymous, and non-coding mutations, we only compared the difference of lipid profiles between patients with missense mutations and frameshift mutations. We found that the TC [5.03 mmol/L (95% CI: 4.82–5.24) vs. 4.52 mmol/L (95% CI: 4.10–4.94), *p* = 0.034] and HDL-c [1.36 mmol/L (95% CI: 1.25–1.47) vs. 1.09 mmol/L (95% CI: 0.98–1.20), *p* < 0.001] levels were significantly higher in patients with missense mutations compared with those with frameshift mutations. Age of diagnosis, FPG, TG, LDL-c, gender, diabetic retinopathy, diabetic neuropathy, and anti-diabetic therapies did not differ by types of *HNF1-alpha* mutations (**Supplementary Table S2**).

### Clinical Manifestations of *HNF1-alpha* MODY by Islet Autoantibody Status

A total of 15 patients from 9 articles were identified to be positive for islet autoantibodies (GAD, IA2, or ICA). Compared with patients with negative islet autoantibodies, patients with positive islet autoantibodies had higher levels of FPG [12.1 mmol/L (95% CI: 9.9–14.2) vs. 8.6 mmol/L (95% CI: 7.8–9.5), *p* = 0.004] and 2h PG [19.7 mmol/L (95% CI: 17.4–21.9) vs. 16.0 mmol/L (95% CI: 15.0–17.0), *p* = 0.003] and lower levels of HDL-c [0.99 mmol/L (95% CI: 0.85–1.13) vs. 1.49 mmol/L (95% CI: 1.30–1.67), *p* < 0.001], and they more often had diabetic neuropathy [50.0% (95% CI: 20.0–80.0) vs. 11.3% (95% CI: 5.7–21.0), *p* = 0.010]. The levels of TG, TC, and LDL-c and the prevalence of macrovascular complications did not differ by islet autoantibody status (**Table 4**).

**Table 4:** Characteristics of patients with *HNF1-alpha* by islet autoantibody status.

Subject	Total (*n* = 1,325)	Negative (*n* = 1,310)	Positive (*n* = 15)	*P*-value
Age of diagnosis (years)	20.1 (16.2, 24.0)	19.8 (15.7, 23.9)	23.4 (18.3, 28.5)	0.282
BMI (kg/m^2^)	22.7 (21.5, 23.9)	22.6 (21.4, 23.9)	23.8 (21.3, 26.3)	0.399
HbA1c (%)	7.7 (7.3, 8.0)	7.6 (7.3, 7.9)	8.9 (7.4, 10.3)	0.094
FPG (mmol/L)	8.9 (8.0, 9.8)	8.6 (7.8, 9.5)	12.1 (9.9, 14.2)	0.004*
2-hour PG (mmol/L)	16.6 (14.5, 18.7)	16.0 (15.0, 17.0)	19.7 (17.4, 21.9)	0.003*
Fasting C-peptide (ng/ml)	1.07 (0.54, 1.60)	1.05 (0.48, 1.63)	1.19 (0.63, 1.74)	0.747
2-h post-load C-peptide (ng/ml)	2.11 (1.61, 2.60)	2.17 (1.59, 2.76)	1.83 (1.26, 2.40)	0.407
TG (mmol/L)	1.12 (0.92, 1.32)	1.12 (0.90, 1.35)	1.14 (0.86, 1.41)	0.942
TC (mmol/L)	4.73 (4.43, 5.04)	4.77 (4.44, 5.10)	4.40 (3.67, 5.13)	0.365
HDL-c (mmol/L)	1.42 (1.24, 1.60)	1.49 (1.30, 1.67)	0.99 (0.85, 1.13)	<0.001*
LDL-c (mmol/L)	2.81 (2.54, 3.08)	2.80 (2.05, 3.10)	2.87 (2.33, 3.41)	0.821
Male (%)	41.1 (34.7, 47.9)	41.5 (34.9, 48.4)	30.8 (12.0, 59.1)	0.450
Family history (%)	87.2 (51.5, 97.8)	88.0 (46.3, 98.4)	86.7 (59.5, 96.6)	0.929
Microvascular complications (%)	48.4 (31.0, 66.2)	47.5 (29.3, 66.3)	62.5 (28.5, 87.5)	0.461
Diabetic retinopathy (%)	20.8 (14.2, 29.4)	20.6 (13.8, 29.7)	25.0 (6.3, 62.3)	0.769
Diabetic kidney disease (%)	15.3 (9.0, 24.7)	16.3 (9.7, 25.9)	100 (0, 100)	1.000
Diabetic neuropathy (%)	12.8 (6.7, 23.3)	11.3 (5.7, 21.0)	50.0 (20.0, 80.0)	0.010*
Macrovascular complications (%)	11.5 (7.7, 16.8)	11.4 (7.5, 17.0)	12.5 (1.7, 53.7)	0.925
Lifestyles (%)	17.0 (13.2, 21.6)	17.2 (13.4, 21.9)	9.1 (1.3, 43.9)	0.490
OHA (%)	39.7 (31.7, 48.3)	40.1 (31.9, 49.0)	27.3 (9.1, 58.6)	0.408
INS (%)	36.2 (31.8, 40.9)	35.8 (31.4, 40.5)	54.6 (26.8, 79.7)	0.213
OHA + INS (%)	9.8 (5.1, 18.0)	9.8 (4.7, 19.3)	9.1 (1.3, 43.9)	0.939
Dimerization domain (%)	5.8 (3.5, 9.6)	5.6 (3.3, 9.4)	11.1 (1.5, 50.0)	0.498
DNA-binding domain (%)	22.5 (8.7, 47.1)	28.7 (13.0, 52.2)	0 (0, 100)	1.000
Transactivation domain (%)	68.3 (47.6, 83.7)	64.1 (42.8, 81.1)	88.9 (50.0, 98.5)	0.193

BMI, body mass index; HbA1c, glycated hemoglobin; FPG, fasting plasma glucose; 2-hour PG, 2-h post-load glucose; TG, triglyceride; TC, total cholesterol; HDL-c, high-density lipoprotein cholesterol; LDL-c, low-density lipoprotein cholesterol; OHA, oral hypoglycemic drugs; INS, insulin; *p < 0.05.

## Discussion

In this study, we analyzed the clinical characteristics of patients with *HNF1-alpha* MODY through literature review and found that the clinical manifestations of *HNF1-alpha* MODY differed by geographical regions, *HNF1-alpha* mutations, and islet autoantibody status. Asian patients with *HNF1-alpha* MODY had a higher HbA1c or 2-h post-load C-peptide levels, lower lipid profile levels, and, less often, macrovascular complications. Patients with transactivation domain mutations were diagnosed at an older age. The levels of 2-h post-load C-peptide, TG, TC, and HDL-c were highest, while the prevalence of diabetic neuropathy was lowest in patients with DNA-binding domain mutations. Patients with non-coding mutations had higher levels of HbA1c or 2h PG and a higher prevalence of diabetic kidney disease but with lower fasting C-peptide levels. In addition, hyperglycemia and diabetic neuropathy were more frequent in patients with positive islet autoantibodies.

The prevalence of *HNF1-alpha* MODY vary among nations and healthcare systems. We thus hypothesized that the clinical manifestations of *HNF1-alpha* MODY could also vary by geographical regions due to diversity in genetic background and environmental factors. In this study, Asian patients exhibited higher HbA1c levels compared with non-Asian patients despite similar fasting and post-load blood glucose. The genetic factors that determine the correlation between HbA1c levels and blood glucose levels could partially explain this discrepancy. In a meta-analysis involving data from 49,238 individuals without diabetes, the HbA1c values are higher in Asians compared to White persons (31). In addition, the postprandial glycemic responses are higher in Asians compared with Caucasians following the ingestion of breakfast cereal and rice (32–35), which could result in higher post-prandial blood glucose and HbA1c levels, but not reflected in post-load glucose levels. In our study, Asian patients with *HNF1-alpha* MODY had a lower BMI but had higher 2-h post-load C-peptide levels. Asians are more likely to have less muscle mass and more visceral fat and are more insulin resistant at a lower BMI (36). The higher levels of 2-h post-load C-peptide in Asian patients may be associated with insulin resistance. In a study involving 94,952 Chinese adults (37), approximately 24.4% of the incidents of diabetes could be attributed to insulin resistance and 12.4% could be attributed to β-cell dysfunction, which, in part, suggested that insulin resistance show a stronger association with incident diabetes than does β-cell dysfunction in Asia. Asian patients with *HNF1-alpha* MODY had lower TG, TC, and HDL-c levels and less often had macrovascular complications despite a higher HbA1c level. The lower lipid profiles among Asians are associated with lifestyle and dietary factors (38). Some single-nucleotide polymorphisms in Asians are also closely related to dyslipidemia, which could independently influence the occurrence of macrovascular complications (39). Age, age of diagnosis, and diabetes duration did not differ between Asian and non-Asian patients with *HNF1-alpha* MODY. Thus, our study suggested that the macrovascular complications of *HNF1-alpha* MODY may be impacted by race and blood lipid. However, a further study, with individual-level patient data, is needed to discern associations between risk factors and macrovascular complications among patients with *HNF1-alpha* MODY. With respect to microvascular complications, in a study involving 667 affected members of *HNF1-alpha* MODY, the prevalence of proliferative retinopathy and proteinuria was 21 and 19%, respectively, higher than *GCK*-MODY and other MODY types (40). The pooled results of our study were similar to the above-mentioned report. Among *HNF1-alpha MODY* patients identified in our study, the prevalence of microvascular diabetic retinopathy was 21.5% (95% CI: 14.5–30.8), and the prevalence of diabetic kidney disease was 16.6% (95% CI: 10.3–25.5). Isomaa B *et al.* found that the risk of microvascular complications in *HNF1-alpha* MODY was closely related to poor glycemic control, diabetes duration, and *HNF1-alpha* mutations (41). In our study, the prevalence of microvascular complications was similar between Asian and non-Asian patients despite the Asian patients having higher HbA1c levels.

*HNF1-alpha*, as a widely expressed tissue-specific transcription factor located at q24.31 on chromosome 12, is composed of 631 amino acids and contains 10 exons (42). In pancreatic β-cells, *HNF1-alpha* regulates the expression of genes associated with β-cell maturation, growth, and function, including glucose transport/metabolism and insulin secretion (43); in the liver, *HNF1-alpha* regulates the expression of tissue-specific regulatory proteins and participates in the metabolism of glucose, fat, and other substances (44); in the kidney, *HNF1-alpha* regulates the expression of SGLT2 and controls glucose reabsorption in proximal tubules (45). The *HNF1-alpha* protein comprises 3 functional domains: dimerization, DNA-binding, and transactivation domains, of which DNA-binding (41.4%) and transactivation domain (55.1%) were predominant in our research. It has been shown that the transactivation domain was more accommodating to mutations causing minor changes in the *HNF1-alpha* protein structure than the dimerization domain or the DNA-binding domain (46) such that mutations in the transactivation domain may not be associated with overt diabetes or a severe phenotype, in line with the observation in our study that the age of diagnosis of patients with transactivation domain mutations was older. However, in the dimerization domain or the DNA-binding domain, there were some crucial sites for the function of *HNF1-alpha* protein, such as exons 1, 4, and 6 (47). Our study also showed that, in patients with dimerization or DNA-binding domain mutations, the age of diagnosis was younger than those with transactivation domain mutations. Moreover, in our study, different types of mutations of *HNF1-alpha* are spread throughout the entire sequence of the gene, including missense mutations, frameshift mutations, nonsense mutations, synonym mutations, and non-coding mutations. In comparison, patients with non-coding mutations had the highest levels of HbA1c or 2h PG and the highest prevalence of diabetic kidney disease but with the lowest levels of BMI or fasting C-peptide among different types of *HNF1-alpha* mutations. The result of this study suggested that more attention is needed for the clinical characteristics of *HNF1-alpha* MODY in different mutations, especially the rare mutations in non-coding regions.

In patients with *HNF1-alpha* MODY, severe hyperglycemia usually occurs before 25–35 years of age and may lead to the misdiagnosis of T1DM. Islet autoantibodies, as an important basis for the diagnosis of T1DM, can be detected in 87–94% of T1DM but are less common in other diabetes (48). In current guidelines for the molecular genetic diagnosis of MODY, absence of islet autoantibodies is one of the criteria for testing for MODY in children and young adults with diabetes and a strong family history of diabetes (49). However, some studies have shown that the islet autoantibodies can be detected in parts of patients with T2DM or MODY and the general population. The initial publication reported that less than 4% of the general population had positive autoantibodies (50). Some studies found islet autoantibodies in 21–33% of children and young people with a clinical diagnosis of T2DM (51, 52). In a study involving 508 patients with MODY (including 229 *HNF1-alpha* MODY patients), GAD positivity, defined as >99th centile of 500 adult control subjects, was detected in 5 patients (<1%, 3 of which were *HNF1-alpha* MODY patients). Among the 5 MODY patients with positive autoantibodies, 4 patients had a clinical course consistent with MODY, while 1 patient was consistent with T1DM (53). In our study, 15 out of 1,325 *HNF1-alpha* MODY patients were found to have positive islet autoantibodies, which was consistent with the above-mentioned reports. Then, we further investigated the special characteristics of patients with positive islet autoantibodies in *HNF1-alpha* MODY. It was noted that patients with positive islet autoantibodies had higher blood glucose levels and more likely had diabetic neuropathy in our study, suggesting that the features of T1DM is present in some MODY patients with positive autoantibodies. In this subset of patients, double diabetes may be the appropriate diagnosis rather than either MODY or T1DM (53). However, there was only approximately 1% of patients with double DM; hence, conducting islet autoantibody testing for all patients with *HNF1-alpha* MODY may be not necessary. Thus, further studies are needed to explore and determine whether islet autoantibody testing is necessary for patients with *HNF1-alpha* MODY.

The intent of this study was to summarize the available published information regarding the clinical characteristics of *HNF1-alpha* MODY. The study has the following limitations: firstly, some studies identified were not included due to the unavailability of key clinical information indicated in the eligible study criteria. Secondly, not all geographic regions were represented in the results—for example, no studies reporting *HNF1-alpha* MODY patients in Africa were identified. Thirdly, the ethnicity of the majority of participants was not specified. Fourthly, due to the limited clinical data in enrolled studies, there was a restriction of further statistical analyses like regression analysis to investigate the association between clinical variables (*e*.*g*., diabetes duration and type of treatment) and chronic complications. More studies with individual-level patient data are needed in the future. Lastly, although we observed more severe clinical characteristics in patients with dimerization/DNA-binding domain mutations or non-coding mutations, further studies are needed to explore the effect of variants in dimerization/DNA-binding domain or non-coding regions for the purpose of understanding the precise molecular mechanism of *HNF1-alpha* MODY.

In conclusion, our study demonstrated that the clinical manifestations of *HNF1-alpha* MODY differed by geographical regions, *HNF1-alpha* mutations, and islet autoantibody status. Asian patients with *HNF1-alpha* MODY had a lower prevalence of macrovascular complications despite higher HbA1c or 2-h post-load C-peptide levels. Patients with transactivation domain mutations were diagnosed at an older age. The levels of 2-h post-load C-peptide, TG, TC, and HDL-c were highest and the prevalence of diabetic neuropathy was lowest in patients with DNA-binding domain mutations. Patients with non-coding mutations had higher levels of HbA1c or 2h PG and a higher prevalence of diabetic kidney disease but with a lower fasting C-peptide level. Hyperglycemia and diabetic neuropathy were more frequent in patients with positive islet autoantibodies.

## Data Availability Statement

The original contributions presented in the study are included in the article/**Supplementary Material**. Further inquiries can be directed to the corresponding authors.

## Author Contributions

QZ and LD researched the data. QZ wrote the manuscript. YY, JS, MW, and XL contributed to the discussion and reviewed/edited the manuscript. ML initiated and designed the research project, reviewed the data, and wrote the manuscript. ML is the guarantor of this work and, as such, had full access to all the data in the study and take responsibility for the integrity of the data and the accuracy of the data analysis. All authors contributed to the article and approved the submitted version.

## Funding

This work was supported by the National Key R&D Program of China (2019YFA0802502) and the National Natural Science Foundation of China (81830025, 81620108004, and 82100865). We acknowledge the support of the Tianjin Municipal Science and Technology Bureau (18JCYBJC93900) and the Tianjin Key Medical Discipline (Specialty) Construction Project.

## Conflict of Interest

The authors declare that the research was conducted in the absence of any commercial or financial relationships that could be construed as a potential conflict of interest.

## Publisher’s Note

All claims expressed in this article are solely those of the authors and do not necessarily represent those of their affiliated organizations, or those of the publisher, the editors and the reviewers. Any product that may be evaluated in this article, or claim that may be made by its manufacturer, is not guaranteed or endorsed by the publisher.
