# Supplementary material for: Clinical Characteristics of Patients With HNF1-alpha MODY: A Literature Review and Retrospective Chart Review
Source: Front Endocrinol (Lausanne). 2022 Jun 20;13:900489. doi: 10.3389/fendo.2022.900489 (PMC9252268; doi:10.3389/fendo.2022.900489)
Supplement: Supplementary file 1 [file Table_1.docx]

Supplementary Material

# Search strategy for database as follows.

# #1 "maturity onset diabetes of the young, type 3"[Supplementary Concept]

# #2 (((((Maturity-Onset Diabetes of the Young, Type 3[Title/Abstract]) OR (MODY 3[Title/Abstract])) OR (MODY3[Title/Abstract])) OR (HNF1A MODY[Title/Abstract])) OR (HNF1-alpha MODY[Title/Abstract])) OR (MODY, type 3[Title/Abstract])

# #3 #1 OR #2

# #4 "hnf1a protein human"[Supplementary Concept] OR "Hepatocyte Nuclear Factor 1-alpha"[MeSH Terms]

# #5 (((((Hepatocyte Nuclear Factor 1-alpha[Title/Abstract]) OR (HNF1A[Title/Abstract])) OR (HNF1-alpha[Title/Abstract])) OR (nuclear protein LF-B1[Title/Abstract])) OR (LF-B1 transcription factor[Title/Abstract])) OR (HNF1 homeobox A protein[Title/Abstract])

# #6 #4 OR #5

# #7 "Diabetes Mellitus"[Mesh]

# #8 diabetes[Title/Abstract]

# #9 #7 OR #8

# #10 #6 AND #9

# #11 #3 OR #10

# Supplementary Tables

**Supplementary Table 1.**

| Articles | Year | Country | Sex | Age of diagnosis (years) | BMI  (kg/m^2^) | Family history | HbA1c (%) | FPG (mmol/L) | PPG (mmol/L) | FC (ng/ml) | PC (ng/ml) | TG  (mmol/L) | TC  (mmol/L) | HDL-c  (mmol/L) | LDL-c  (mmol/L) | Complications | Treatment | Mutation |
| --- | --- | --- | --- | --- | --- | --- | --- | --- | --- | --- | --- | --- | --- | --- | --- | --- | --- | --- |
| Xiaoyan, Ren, et al(1) | 2021 | China | male | 12 | 17.7 | yes |  | 7.4 | 18.5 | 1.32 | 2.76 | 0.83 | 5.08 | 1.14 | 2.61 | None | OHA | c.G392T/p.R131L |
|  |  |  | female | 24 |  | yes |  | 8 |  |  |  |  |  |  |  |  | Lifestyles |  |
| Mengyan, Xu, et al(2) | 2018 | China | Female | 35 | 20.83 | yes | 6.5 | 5.2 | 13.6 | 2.09 | 3.85 | 0.42 | 4.08 | 1.60 | 2.29 | none | OHA | c.512G>A/p.Arg171Gln |
|  |  |  | Female | 17 | 19.53 | yes | 10.2 | 8.5 | 20.4 | 1.75 | 4.04 | 1.15 | 3.98 | 1.28 | 2.98 | none | OHA | c.391C>T/p.Arg131Trp |
|  |  |  | Male | 35 |  | yes |  |  |  |  |  |  |  |  |  | DR | OHA | c.512G>A/p.Arg131Gln |
|  |  |  | Male | 37 |  | yes |  |  |  |  |  |  |  |  |  |  | OHA | c.391C>T/p.Arg131Trp |
|  |  |  | Male | 40 |  | yes |  |  |  |  |  |  |  |  |  |  | OHA |  |
|  |  |  | Male | 24 |  | yes |  |  |  |  |  |  |  |  |  | DR, DKD | OHA+INS |  |
| Xiaoli Kang, et al(3) | 2017 | China | female | 13 | 21.8 | yes | 10.8 | 16.82 | 28.39 | 1.10 | 2.71 |  |  |  |  | none |  | c.51C>G/p.Leu17Leu |
|  |  |  | female | 23 |  | yes |  |  | 12.6 |  |  |  |  |  |  |  | OHA | c.79A>C/p.Ile27Leu |
|  |  |  | female | 48 |  | yes |  |  |  |  |  |  |  |  |  |  | OHA | c.814C>A/p.Arg272Ser |
|  |  |  | female | 26 |  | yes |  |  |  |  |  |  |  |  |  | DN, DR | OHA | c.1375C>T/p.Leu459Leu |
|  |  |  | female | 14 |  | yes |  |  |  |  |  |  |  |  |  |  | OHA | c.1460G>A/p.Ser487Asn |
|  |  |  | female | 11 |  | yes | 10.8 |  |  |  |  |  |  |  |  | DR | INS | c.326+91A>G/IVSInt+91A>G |
|  |  |  | male | 16 |  | yes |  |  | 18.2 |  |  |  |  |  |  |  |  | c.1107+9C>G/IVS5nt+9C>G |
|  |  |  | female | 16 |  | yes | 10.1 | 16.82 | 23.21 |  |  |  |  |  | 3.38 |  |  | c.1501+7G>A/IVS7nt+7G>A |
| Liubov Desiatkina, et al(4) | 2015 | china | male | 19 | 26.85 | yes | 12.3 | 17.6 | 27 | 1.42 | 1.91 |  |  |  |  |  | OHA | c.1410G>A/p.S487N |
|  |  |  | female | 52 |  | yes | 8.3 | 8 | 12 |  |  |  |  |  |  |  | INS |  |
| Tianping Wang, et al(5) | 2014 | china | female | 17 | 18.7 | yes | 5.4 | 4.94 |  | 0.70 | 2.44 | 0.77 | 4.0 | 1.20 | 2.45 | DN | INS | c.1130-1131insC/p.V380cfs*39 |
|  |  |  | female | 36 | 19.6 | yes | 6.3 | 6.11 | 10.2 | 0.92 | 3.61 | 1.11 | 2.60 | 0.47 | 1.625 | DN,DR | lifestyles |  |
|  |  |  | female | 24 | 17.3 | yes | 9.2 | 10.88 |  | 0.92 | 1.80 | 0.84 | 4.40 | 0.99 | 3.028 | DN,DR | OHA |  |
| Xin pan,et al(6) | 2021 | china | female | 22 |  |  | 7.5 | 9.79 | 23.03 | 2.32 | 4.36 | 0.8 | 3.84 | 0.93 | 2.59 | none | OHA | p.P379T |
| Yuecheng Zhang, et al(7) | 2015 | china | female | 14 | 16.3 | yes | 5.7 | 6.2 | 6.8 |  |  | 1.65 |  |  |  | none |  | c.293C>T/p.X466W |
| Yipaerguli．Ainiwaner, et al(8) | 2021 | china | male | 6 | 16.5 | yes | 6.5 | 6.13 | 12.83 | 0.6 | 2.45 |  |  |  |  | none | OHA | c.A1398G/p.X466W |
|  |  |  | female | 46 | 20.24 | yes | 10.8 | 13.7 | 18.52 | 1.43 |  |  |  |  |  | none | OHA+INS |  |
|  |  |  | male | 30 | 21.22 | yes | 7.2 | 7.72 | 10.39 | 2.2 |  |  |  |  |  | none | OHA |  |
|  |  |  | male | 75 | 24.22 | yes |  | 12.29 | 17.83 |  |  |  |  |  |  | Coronary heart disease | OHA+INS |  |
|  |  |  | female | 48 | 27.29 | yes | 6.9 | 5.58 | 11.68 |  |  |  |  |  |  | none | OHA+INS |  |
| Yanwen Shu, et al(9) | 2021 | china | female | 26 | 20.47 | no | 12 |  | 17.4 | 0.63 | 2.13 | 1.15 |  | 1.01 | 2.55 | DR, DKD, DN | INS | c.865dupC/p.G292Rfs*25 |
|  |  |  | female | 21 | 26.31 | yes | 5.9 |  | 14 | 2.23 | 8.09 | 4.24 |  | 0.88 | 2.09 | none | OHA | c.961C>A/p.L108P |
|  |  |  | female | 12 | 19.74 | yes | 12.1 |  |  | 1.95 | 3.21 | 1.28 |  | 0.92 | 1.34 | none | OHA | c.323T>G/p.L108P |
|  |  |  | female | 43 | 18.8 | yes | 6.5 |  |  |  |  |  |  |  |  | none | OHA |  |
| Mingwei Shao, et al(10) | 2020 | china | male | 12 | 20.8 | yes | 9.3 | 7.4 | 17.3 |  |  |  |  | 1.49 |  |  | OHA | c.779C>T/p.Thr260Met |
|  |  |  | male | 38 | 22.39 | yes | 9.4 | 12.4 | 26.8 |  |  |  |  |  |  |  | OHA |  |
| Ying Zhang, et al(11) | 2021 | china | female | 19 | 23.1 | yes | 9.6 | 11.2 | 20.6 | 0.05 | 3.34 |  |  |  |  | DR, DN | OHA | c.G812A/p.R271Q |
| Nikolai Paul Pace, et al(12) | 2019 | Malta | female | 14 | 19.56 | yes | 9.9 |  |  | 2.1 |  | 1.95 | 4.44 | 0.82 | 2.73 |  | INS | c.872dup/p.Gly2992fs |
|  |  |  | female | 16 | 23.32 | yes | 7.0 | 5.67 | 14.21 | 1.6 |  | 1.17 | 3.08 | 0.40 | 2.15 |  | INS |  |
|  |  |  | female | 19 | 22.65 | yes | 8.3 | 8.64 | 17.24 | 2.8 |  | 0.63 | 4.57 | 1.91 | 2.37 |  | INS |  |
| Pedro J, et al(13) | 2011 | Spain | Male11/female 4 | 17.6±4.4 | 26.0±4.2 | 2 patients | 7.4±1.5 |  |  |  |  |  |  | 2.90±0.90 |  |  | OHA 6/INS 3/OHA+OMNS 2 |  |
| Fernando M.A. et al(14) | 2016 | Brazil | Male 11/female 20 | 24±12 | 22±4 | yes | 7.0±1.4 | 7.8±3.1 |  | 0.5±0.2 |  | 9.2±3.6 | 5.0±0.9 | 1.4±0.3 | 3.1±0.7 |  | OHA 28/INS 5 |  |
| Maja D. Jesic, et al(15) | 2008 | Serbia | female | 10 | 20 | yes | 7.9 | 8.7 | 16.2 |  |  | 1.70 | 3.96 | 0.72 | 2.47 | Peripheral arterial disease | OHA | c.368T>C/p.Leu123Pro |
|  |  |  | female | 14 |  | yes |  |  |  |  |  |  |  |  |  |  | OHA |  |
| Junling Fu, et al(16) | 2019 | china | Total 12 | 21.75±8.74 | 20.70±1.65 |  | 8.31±2.62 | 8.26±3.17 | 13.46±6.34 | 1.34±1.51 | 2.42±1.35 | 0.97±0.,43 | 4.41±0.93 | 1.32±0.39 | 2.57±0.86 |  |  |  |
| N. Tonooka, et al(17) | 2002 | japan | Male 6/female 12 | 11.6±1.7 | 20.1±3.0 | 1 patients |  |  |  |  |  |  |  |  |  | none | Lifestyles 4/OHA 5/INS 9 |  |
| W L Awa, et al(18) | 2011 | Austria | Male 16/female 28 | 14.1±5.8 |  | 21 patients | 6.8±1.2 |  |  |  |  |  |  |  |  |  | Lifestyles 9/INS 16/OHA+INS 10 |  |
| Birgit Knebel, et al(19) | 2016 | Germany | female | 43 | 27 | yes | 7.4 | 12.7 | 22.2 | 0.4 | 0.6 | 2.2 | 7.0 | 1.1 | 4.8 | none |  | c.1785C>T/p.Pro588Ser  c.1765-1766delCA |
|  |  |  | male | 39 | 30 | yes | 8.9 | 12.8 | 16.0 | 0.7 | 1.2 | 6.9 | 7.3 | 0.7 | 3.4 | none |  |  |
| H.C.Fehmann, et al(20) | 2003 | Germany | male | 16 |  | yes | 6.4 | 8.0 | 13.0 |  |  |  |  |  |  |  |  | c.224C>T/p.Peo224Ser |
|  |  |  | female | 25 |  | yes |  |  |  |  |  |  |  |  |  |  |  |  |
| S. Bacon, et al(21) | 2015 | England | Male 24/female 35 |  |  |  |  |  |  |  |  |  |  |  |  | DR 10/DKD 6/Coronary heart disease 4 | Lifestyles 15/ OHA 21/INS 15 |  |
| Aoife M Egan, et al(22) | 2015 | England | female | 12 |  | yes | 12.7 | 10 | 23 | 1.5 |  |  |  |  |  | DR, DKD | INS | c.391C>T/p.Arg131Trp |
| Rachel E J Besser, et al(23) | 2012 | England | female | 14 |  | yes | 9.3 | 9.3 |  |  |  |  |  |  |  | Coronary heart disease | OHA | c.608G>C/p.R203H |
|  |  |  | male | 30 | 30 | yes | 9.7 |  |  |  |  |  |  |  |  |  |  |  |
| Shivani Misra, et al(24) | 2020 | England | male | 15 | 27.8 | yes | 7.5 |  | 7.4 | 0.6 |  |  |  |  |  |  | INS | P.A251T/A251T |
|  |  |  | male | 12 | 28.7 | yes | 8.3 |  | 10.9 | 0.79 |  |  |  |  |  |  | INS |  |
|  |  |  | female | 16 | 23.2 | yes | 12.2 |  | 7 | 0.47 |  |  |  |  |  |  | INS |  |
|  |  |  | female | 29 | 25.4 | yes | 8.7 |  | 13.8 | 1.51 |  |  |  |  |  |  | OHA | p.A251T/N |
| Maricor K, et al(25) | 2013 | The USA | female | 10 | 29.6 | yes |  | 10.67 |  | 0.8 |  |  |  |  |  | DKD, DN | OHA+INS | c.598C>T/p.Arg200Trp |
|  |  |  | female | 9 | 30.8 | yes | 7.7 | 13.28 |  | 0.5 |  |  |  |  |  |  | INS |  |
|  |  |  | female | 10 | 32.5 | yes | 9.0 | 11.17 |  | 0.8 |  |  |  |  |  |  | INS |  |
| Yael Lebenthal, et al(26) | 2017 | England | female | 24 | 24 | no |  |  |  |  |  |  |  |  |  | DR | OHA |  |
|  |  |  | female | 16 | 22.5 | no |  |  |  |  |  |  |  |  |  | DR, DKD | OHA+INS |  |
|  |  |  | female | 11 | 21 | no | 7.9 |  |  |  |  |  |  |  |  |  | INS |  |
|  |  |  | female | 10 | 25.6 | no | 6.3 |  |  |  |  |  |  |  |  | DKD | INS |  |
|  |  |  | male | 21 | 21.8 | no |  |  |  |  |  |  |  |  |  | DR | OHA+INS |  |
|  |  |  | male | 50 | 19.5 | yes |  |  |  |  |  |  |  |  |  |  | OHA |  |
|  |  |  | female | 23 | 27.3 | no |  |  |  |  |  |  |  |  |  | DR | OHA+INS |  |
|  |  |  | male | 22 | 21.5 | no | 7.8 |  |  |  |  |  |  |  |  |  | INS |  |
|  |  |  | male | 17 | 21.5 | no | 6.7 |  |  |  |  |  |  |  |  |  | INS |  |
|  |  |  | male | 14 | 23.1 | yes | 6.7 |  |  |  |  |  |  |  |  | DKD | OHA+INS |  |
|  |  |  | male | 45 | 22.7 | no |  |  |  |  |  |  |  |  |  |  | OHA |  |
|  |  |  | female | 49 | 21.9 | no | 7.1 |  |  |  |  |  |  |  |  |  | OHA |  |
|  |  |  | female | 10 | 27 | yes | 9.4 |  |  |  |  |  |  |  |  |  | INS |  |
|  |  |  | male | 13 | 24.2 | no | 8.1 |  |  |  |  |  |  |  |  |  | OHA+INS |  |
|  |  |  | female | 13 | 28.5 | yes | 8.9 |  |  |  |  |  |  |  |  |  | OHA+INS |  |
| Souhaïra Ben Khelifa, et al(27) | 2015 | Tunisia | female | 14 | 25.49 | yes | 10.8 | 11.23 |  | 0.52 |  | 1.03 | 4.92 | 1.24 |  | DKD | OHA | P291fsinsC |
| Magdalena Szopa, et al(28) | 2015 | Poland | Male 26/female 46 | 24.95±10.96 | 25.05±4.0 |  | 6.83±1.41 | 6.91±2.68 |  | 1.33±0.66 |  | 1.23±0.92 | 4.79±0.97 | 1.59±0.42 | 2.64±0.86 |  |  |  |
| K. A. Iwen, et al(29) | 2012 | Germany | female | 20 |  | yes | 6.3 | 4.5 | 16.9 |  |  |  |  |  |  |  | OHA | PA98V polymorphism + Q495X stop |
| Atsushi Iwabuchi, et al(30) | 2013 | Japan | male | 1 |  | yes | 7.8 | 7.7 | 18.7 |  |  |  |  |  |  |  | OHA | c.593delA/p.Lys198fs |
| B.Isomaa, et al(31) | 1998 | Finland | Male 29/female 28 | 26.7±2.9 | 24.6±4.5 |  | 7.3±1.6 |  |  |  |  | 1.53±0.82 | 5.3±1.0 | 1.40±0.21 |  | DR 18/DKD 19/DN 17/Coronary heart disease 9 | Lifestyles 19/OHA 17/INS 21 |  |
| M. Hummel, et al(32) | 2006 | Germany | female | 17 | 20.3 | yes | 7.2 |  |  | 1.2 |  |  |  |  |  | none | OHA+INS | c.653A>G/p.Tyr218Cys |
|  |  | Belgium | male | 18 | 21.9 | yes |  |  |  | 1 |  |  |  |  |  |  | INS |  |
|  |  |  | female | 15 | 18.1 | yes |  |  |  | 1.19 |  |  |  |  |  |  | INS |  |
| Itziar Estalella, et al(33) | 2007 | Spain | Total 24 | 12.7±4.6 |  |  | 7.6±2.3 | 11.3±3.2 |  |  |  |  |  |  |  |  | Lifestyles 4/OHA 1/INS 3 |  |
| Naieli Bonatto, et al(34) | 2012 | Brazil | female | 46 | 28.37 | yes | 6.4 | 9.17 | 10.33 | 2.52 |  |  |  |  |  | Microvascular and macrovascular complications | INS |  |
|  |  |  | female | 36 | 30.30 | yes | 14.3 | 15.61 | 20.22 | 3.37 |  |  |  |  |  | none | INS |  |
|  |  |  | male | 26 | 26.47 | yes | 9.78 | 22.56 | 18.44 | 1.40 |  |  |  |  |  | none | INS |  |
|  |  |  | male | 38 | 33.57 | yes | 10.68 | 15.44 | 23.33 | 3.50 |  |  |  |  |  | none | OHA |  |
|  |  |  | female | 16 | 33.30 | yes | 11.3 | 14.44 | 22.11 | 1.20 |  |  |  |  |  | Microvascular and macrovascular complications | INS |  |
|  |  |  | female | 40 | 28.58 | yes | 11.45 | 16.94 | 18.11 | 2.10 |  |  |  |  |  | macrovascular complications | OHA |  |
|  |  |  | male | 45 | 31.94 | yes | 12.6 | 16.72 | 18 | 1.20 |  |  |  |  |  | Microvascular complications | OHA+INS |  |
| Wojciech Fendler, et al(35) | 2011 | poland | Male 26/female 11 |  |  |  |  |  |  |  |  |  |  |  |  |  | Lifestyles 2/OHA 16/INS 19 |  |
| Chen Fang, et al(36) | 2015 | china | female | 19 | 22 | yes | 15 | 18 | 21.56 | 1.48 |  |  |  |  |  |  | OHA | R45X (CGA>TGA) |
| Nattachet Plengvidhya, et al(37) | 2019 | Thailand | female | 14 | 19.43 | yes | 6.7 | 7 |  |  |  | 2.97 | 8.43 | 3.84 |  | none | OHA | R203C |
| Letı´cia S, et al(38) | 2014 | Brazil | male | 23 | 24.7 | yes | 7.2 |  |  | 1.6 |  |  |  |  |  | DR, DKD | OHA | c.24-35dup12/p.Gln9-Leu12du |
|  |  |  | female | 27 | 19.2 | yes | 6.7 |  |  | 1.6 |  |  |  |  |  | none | OHA+INS | c.872dupC/p.Gly292fs |
| Xiaojing Wang, et al(39) | 2018 | china | Male 12/female 5 | 25.56±13.32 | 20.91±2.23 |  | 8.36±2.14 | 8.75±2.93 | 16.19±6.08 | 0.91±0.40 | 1.77±0.93 |  | 4.53±1.02 |  | 2.75±0.86 |  |  |  |
| Elizabeth B. Tatsi, et al(40) | 2019 | Greece | female | 13.5 | 21 | yes | 11.5 |  |  |  |  |  |  |  |  |  |  | c.1204A>T/p.Asn402Tyr |
|  |  |  | female | 12.5 | 22.8 | yes | 7.4 |  |  |  |  |  |  |  |  |  |  | c.599G>A/p.Arg200Gln |
|  |  |  | male | 30 |  | no |  |  |  |  |  |  |  |  |  |  |  |  |
| M. Szopa, et al(41) | 2015 | Poland | Male 47/female 16 | 23.5±9.8 | 23.9±3.8 |  | 7.0±1.4 | 7.3±2.8 |  | 1.3±0.7 |  | 1.2±0.9 | 4.8±0.9 | 1.6±0.4 | 2.6±0.8 | DR 15/DKD 36 | Lifestyles 4/OHA 35/INS 22 |  |
| J. Skupie, et al(42) | 2008 | Poland | Male 11/female 31 | 24.5±10.9 | 23.0±3.2 |  | 7.5±1.8 | 7.8±3.1 |  | 1.45±0.47 |  | 1.2±0.7 | 5.0±1.1 | 1.6±0.5 | 2.9±0.9 | DR 21/DKD 11/Coronary heart disease 4 | INS 23 |  |
| Ibrar Rafique, et al(43) | 2021 | Pakistan | male | 17 | 24.6 | yes | 8.2 |  |  |  |  |  |  |  |  |  |  | c.526+1G>A |
| Junnosuke Miura, et al(44) | 1997 | japan | female | 19 | 22 | yes | 14.0 | 13.83 |  |  |  |  |  |  |  |  | INS |  |
| Ariel Pablo Lopez, et al(45) | 2010 | Argentina | Total 8 | 20.86 | 23±1.5 |  | 7.5±1.6 | 11.39±2.24 |  |  |  |  |  |  |  |  | Lifestyles 0/OHA 8/INS 0 |  |
| Tim J. McDonald, et al(46) | 2012 | England | Male 41/female 226 |  | 24.9±4.6 |  | 7.5±1.6 |  |  |  |  |  | 4.9±1.3 | 1.46±0.48 |  |  |  |  |
| STEPANKA PRUHOVA, et al(47) | 2013 | Czech Republic | female | 4 | 20.1 |  | 15 |  |  |  |  |  |  |  |  |  | INS | p.Arg272His |
|  |  |  | male | 13 | 29.4 | yes | 13 | 34 |  |  |  |  |  |  |  |  | INS | p.Ser142Phe |
| TIINAMAIJA TUOMI, et al(48) | 2006 | Sweden | female | 23.9 | 25.0 |  | 5.4 | 4.3 |  |  |  |  |  |  |  |  | none |  |
|  |  |  | female | 44.9 | 22.4 |  | 6.6 | 7.0 |  |  |  |  |  |  |  |  | none |  |
|  |  |  | female | 30.8 | 22.6 |  | 8.1 | 10.8 |  |  |  |  |  |  |  |  | INS |  |
|  |  |  | male | 34.2 | 21.9 |  | 6.4 | 7.0 |  |  |  |  |  |  |  |  | OHA |  |
|  |  |  | male | 42.5 | 24.3 |  | 8.7 | 12.2 |  |  |  |  |  |  |  |  | OHA |  |
|  |  |  | female | 50.6 | 23.4 |  | 9.1 | 11.3 |  |  |  |  |  |  |  |  | OHA |  |
|  |  |  | female | 41.1 | 28.2 |  | 6.7 | 5.3 |  |  |  |  |  |  |  |  | none |  |
|  |  |  | female | 62.6 | 24.7 |  | 6.5 | 5.2 |  |  |  |  |  |  |  |  | OHA |  |
|  |  |  | male | 32.9 | 22.3 |  | 7.8 | 7.5 |  |  |  |  |  |  |  |  | none |  |
|  |  |  | male | 46.5 | 23.9 |  | 7 | 7.9 |  |  |  |  |  |  |  |  | none |  |
|  |  |  | male | 66.1 | 23.4 |  | 9.9 | 16.2 |  |  |  |  |  |  |  |  | OHA |  |
|  |  |  | female | 40.2 | 16.7 |  | 5.9 | 8.3 |  |  |  |  |  |  |  |  | OHA |  |
|  |  |  | female | 47.3 | 19.8 |  | 6.1 | 9.4 |  |  |  |  |  |  |  |  | OHA |  |
|  |  |  | female | 20.0 | 23.1 |  | 9 | 5.4 |  |  |  |  |  |  |  |  | OHA |  |
|  |  |  | female | 28.0 | 21.2 |  | 6.7 | 5.0 |  |  |  |  |  |  |  |  | none |  |
| A. K. Ovsyannikova, et al(49) | 2018 | Russia | female | 12 | 24.5 | yes | 7.7 |  |  | 1.01 |  |  |  |  | 3.4 | DR, DN |  | p.Ser6Arg |
|  |  |  | male | 21 | 22.4 | yes | 7.1 | 9 | 12.8 | 0.78 |  |  |  |  | 3.2 | none | OHA |  |
|  |  |  | female | 45 |  |  |  |  |  |  |  |  |  |  |  | DN | OHA |  |
| S Mongolu, et al(50) | 2009 | England | female | 27 |  | yes | 9.8 |  |  |  |  |  |  |  |  |  | OHA |  |
| Wolfgang J. Schnedl, et al(51) | 2020 | Austria | female | 18 | 23 | yes | 8.4 |  |  |  |  |  |  |  | 8.05 |  | OHA | c.815G>A/p.Arg272His |
|  |  |  | female | 41 | 22 | yes |  | 9.19 |  |  |  |  |  |  | 6 |  | OHA | c.675delC/p.Ser225Argfs*8 |
| Giuseppina Salzano, et al(52) | 2019 | Italy | female | 10 |  | no | 10.8 | 21.4 |  | 1.07 | 2.52 |  |  |  |  | none | OHA | c.709A>G |
| Marianne Becker, et al(53) | 2014 | Germany | female | 12 | 23.7 | yes | 7.4 |  |  |  |  |  |  |  |  | none | OHA | c.872dupC/p.Gly292ArgfsX25 |
|  |  |  | male | 14 | 19.2 |  | 7.0 |  |  |  |  |  |  |  |  | none | OHA | c.162T>C/p.Leu54Pro |
| Abdelhadi M. Habeb, et al(54) | 2011 | England | male | 7 |  | yes | 7.8 | 7.7 | 18.7 |  |  |  |  |  |  |  | OHA | c.526+1G>A |
| Maltoni G, et al(55) | 2012 | Italy | male | 15 |  | yes | 7.2 | 6.33 | 14.22 | 1.9 |  |  |  |  |  |  | OHA | c.92G>A/p.G31D |
| Akie Nakamura, et al(56) | 2012 | japan | female | 9 | 15.9 |  | 7.3 | 5.78 | 18.17 |  |  | 7.68 | 11.19 | 2.43 |  |  | INS |  |
| MC Ng, et al(57) | 2000 | china | female | 19 | 19.6 | yes | 8.0 |  |  | 0.47 |  |  |  |  |  | DR,DKD | INS | IVS2nt-1G>A |
|  |  |  | female | 24 | 22.7 | yes | 6.4 |  |  | 0.86 |  |  |  |  |  | DR. DKD | INS |  |
|  |  |  | female | 38 | 18.5 | yes | 17.2 |  |  | 0.32 |  |  |  |  |  | DR, DKD, DN, Coronary heart disease | INS |  |
|  |  |  | female | 12 | 18.8 | yes | 8.8 |  |  | 0.13 |  |  |  |  |  |  | INS |  |
|  |  |  | male | 15 | 16.6 | yes | 5.3 |  |  | 0.36 |  |  |  |  |  |  | INS |  |
| Jose Bernardo Quintos, et al(58) | 2013 | The USA | female | 13 | 21 | yes | 8.5 | 10.72 |  |  |  | 4 | 9.95 | 1.89 | 7.24 |  | OHA | c.1129delC/p.Leu377fsX7 |
| MP Selwood, et al(59) | 2008 | England | female | 25 |  | yes |  |  |  | 0.57 |  |  |  |  |  | DR, DKD, DN |  |  |
|  |  |  | female | 37 | 26.2 | yes | 7.2 | 9.4 |  |  |  |  |  |  |  | none | OHA |  |
|  |  |  | male | 26 |  | yes | 6.4 | 8.0 | 13.0 |  |  |  |  |  |  | none | Lifestyles |  |
| NAOKO IWASAKI, et al(60) | 1998 | japan | Male 6/female 5 | 16.1±2.0 | 20.6±0.4 |  |  |  |  |  |  | 1.53±0.82 | 5.3±1.0 | 1.40±0.21 |  | DR 5/DKD 4 | Lifestyles 2/OHA 3/INS 6 |  |
| M.P. Kyithar, et al(61) | 2010 | Ireland | Male 13/female 18 | 25±2 | 24.52±0.69 |  | 7.21±0.22 | 7.34±0.43 |  | 2.02±0.36 |  | 0.91±0.12 | 4.34±0.19 | 1.42±0.09 | 2.56±0.15 |  | Lifestyles 0/OHA 13/INS 8 |  |
| ALESSANDRO DORIA, et al(62) | 1999 | Poland | Male 32/female 47 | 21±10 |  |  | 6.9±1.6 | 6.44±2.06 | 14.39±5.39 |  |  | 5.41±3.14 | 10.54±2.22 | 2.86±0.76 |  | DR 5/DKD 14/Coronary heart disease 14 | Lifestyles 24/OHA 18/INS 37 |  |
| Miao Zhang, et al(63) | 2015 | china | female | 36 | 19.6 | yes | 6.3 | 6.11 | 10.12 | 0.31 | 1.20 | 1.11 | 2.60 | 0.47 | 1.625 |  | OHA | p.379fsinsC |
|  |  |  | female | 24 | 17.3 | yes | 9.2 | 10.88 |  | 0.31 | 0.60 | 0.84 | 4.40 | 0.99 | 3.028 | DR, DN |  |  |
|  |  |  | female | 23 | 18.7 | yes | 5.4 | 7.76 | 19.84 | 0.24 | 0.69 | 0.77 | 4.00 | 1.20 | 2.45 | DR, DN | OHA |  |
|  |  |  | female | 11 | 19.6 | yes | 7.3 | 5.88 | 13.37 | 0.53 | 1.25 | 0.85 | 4.15 | 1.52 | 2.24 | DN |  |  |
| Nattachet Plengvidhya, et al(64) | 2009 | Thailand |  | 31 | 24.49 |  | 6.60 | 6.94 |  | 0.3 |  | 0.76 | 5.10 | 1.26 | 3.49 |  |  | P475L |
|  |  |  |  | 12 | 20.41 |  | 4.90 | 9.27 |  | 0.66 |  | 0.43 | 3.55 | 1.58 | 1.77 |  |  | G554SfsX556 |
| S. Pruhova, et al(65) | 2003 | Czech Republic | Male 3/female 4 |  |  |  |  |  |  |  |  |  |  |  |  | DR 1/DKD 1/DN 1 | Lifestyles 2/OHA 1/INS 4 |  |
| Juraj Stanik, et al(66) | 2014 | Czech Republic |  | 16 | 20.2 |  | 6.8 | 5.4 |  | 1.29 |  |  |  |  |  |  | INS | c.392G>A/R131Q |
|  |  |  |  | 14 | 22.9 |  | 7.3 | 12.0 |  | 0.59 |  |  |  |  |  |  | INS |  |
|  |  |  |  | 15 | 18.0 |  | 5.9 | 5.4 |  | 0.79 |  |  |  |  |  |  | Lifestyles | c.392G>C/R131P |
|  |  |  |  | 11 | 19.9 |  | 6.2 | 6.2 |  | 1.41 |  |  |  |  |  |  | INS | c.436C>G/Q146E |
|  |  |  |  | 15 | 22.0 |  | 8.6 | 5.8 |  | 1.33 |  |  |  |  |  |  | INS | c.1256C>G/S419* |
| MACIEJ T. MALECKI, et al(67) | 2005 | Poland | female | 17 |  | yes |  |  |  |  |  |  |  |  |  | DR, DKD, macrovascular complications, | INS | IVS7nt-6G>A |
|  |  |  | female |  |  | yes |  |  |  |  |  |  |  |  |  |  | INS | Arg271Trp |
| GAYA THANABALASINGHAM, et al(68) | 2012 | England | Male | 13 | 23.4 | yes |  | 21.3 |  | 1`.08 |  |  |  |  |  |  |  | c.92G>A/G31D |
|  |  |  | female | 29 | 22.5 | no |  | 11.2 |  | 0.50 |  |  |  |  |  |  |  | c.1469T>G/M490R |
| Maraschin et al.(69) | 2008 | Poland | Male 2/female 8 | 21±5 | 24±3 | 10 patients | 7.06±1.6 | 6.9±0.9 |  |  |  | 4.38(2.22-7.03) | 10.6±2 | 3.14±0.4 |  | DR 4/DKD 2/DN 1/Coronary heart disease3 |  |  |
| Catherine Pihoker, et al(70) | 2013 | The USA | Male 7/female 19 | 12.2±3.0 | 26.7±6.8 | 16 patients | 6.9±1.7 |  |  | 2.3±1.2 |  |  |  |  |  |  | Lifestyles 2/OHA 17/INS 15 |  |
| Jian Yu Xu, et al(71) | 2005 | china | Male 9/female 12 | 21.24±4.71 | 22.59±3.45 | 21 patients | 7.47±1.17 | 6.7(5.4-10) |  | 0.26(0.20-0.48) | 1.74(1.16-2.87) | 0.8  (0.63-1.2) | 4.72±0.79 | 1.4(1.1-1.63) | 2.84±0.62 | DR 4/DKD 1/ DN 1/Coronary heart disease 0 | Lifestyles 5/OHA 11/INS 5 |  |
| I. Yoshiuchi, et al(72) | 1999 | japan | female | 25 | 22.2 |  | 7.6 |  |  |  |  |  |  |  |  | DR | INS | G415R |
|  |  |  | male | 30 | 17.3 | yes |  |  |  |  |  |  |  |  |  | DR, DKD, DN | OHA | R272C |
|  |  |  | male | 29 |  | yes | 6.3 |  |  |  |  |  |  |  |  | none | OHA+INS | A site mutation +102G>C |
| R.D. Cox, et al(73) | 1999 | England |  | 42 | 25 | yes |  | 6.2 |  |  |  |  |  |  |  |  | Lifestyles | Promoter -237G>A/G237A |
|  |  |  |  | 33 | 22 | no |  | 7.2 |  |  |  |  |  |  |  |  | Lifestyles | Promoter -8-9 2bp deletion |
|  |  |  |  | 45 | 25 | no |  | 7.2 |  |  |  |  |  |  |  |  | INS | A301T |
|  |  |  |  | 37 | 23 | no |  | 8 |  |  |  |  |  |  |  |  | OHA | T492I |
|  |  |  |  | 46 | 38 | yes |  | 13 |  |  |  |  |  |  |  |  | OHA | S498R |
| S. Demol, et al(74) | 2014 | Israel | male | 13.5 | 32.6 |  | 6.5 | 5.94 |  |  |  |  |  |  |  |  | INS | c.618G>A/p.W206X |
|  |  |  | male | 30 | 26.6 |  | 5.1 | 5.44 |  |  |  |  |  |  |  |  | INS |  |
|  |  |  | female | 19 | 28 |  | 7.5 | 7.83 |  |  |  |  |  |  |  |  | INS |  |
|  |  |  | female | 10.5 | 33.6 |  | 11.1 | 7.78 |  |  |  |  |  |  |  |  | INS |  |
|  |  |  | female |  | 21.1 |  | 5.1 | 5.17 |  |  |  |  |  |  |  |  | none |  |
|  |  |  | male |  | 28.3 |  | 5.3 | 5.44 |  |  |  |  |  |  |  |  | none |  |
|  |  |  | female |  | 20.1 |  | 4.8 | 5.22 |  |  |  |  |  |  |  |  | none |  |
|  |  |  | male |  | 20.8 |  | 5.4 | 5.5 |  |  |  |  |  |  |  |  | none |  |
| Aarón Domínguez-López, et al(75) | 2005 | Mexico | female | 12 | 27.3 | yes |  |  | 22.2 |  |  |  |  |  |  | none | none |  |
| Timothy M. Frayling, et al(76) | 2001 | England | Total 1 | 21 |  | no |  |  |  |  |  |  |  |  |  |  | OHA 1 | P129T |
|  |  |  | Total 3 | 18.7(9-31) |  | yes |  |  |  |  |  |  |  |  |  |  | OHA 2/INS 1 | R131W |
|  |  |  | Total 2 | 15.5(11-20) |  | yes |  |  |  |  |  |  |  |  |  |  | OHA 1/INS1 | R159W |
|  |  |  | Total10 | 20.4(14-38) |  | yes |  |  |  |  |  |  |  |  |  |  | Lifestyles 3/OHA3/ INS 4 | P291fsinsC |
|  |  |  | Total 4 | 27.5)12-70) |  | yes |  |  |  |  |  |  |  |  |  |  | OHA 1/INS 3 |  |
|  |  |  | Total 3 | 17(14-23) |  | yes |  |  |  |  |  |  |  |  |  |  | Lifestyles 1/OHA 1/INS 1 |  |
|  |  |  | Total 1 | 21 |  | no |  |  |  |  |  |  |  |  |  |  | OHA 1 |  |
|  |  |  | Total 7 | 27.4(13-37) |  | yes |  |  |  |  |  |  |  |  |  |  | Lifestyles 3/OHA 2/INS 2 |  |
|  |  |  | Total 3 | 22(8-45) |  | yes |  |  |  |  |  |  |  |  |  |  | Lifestyles 1/OHA 2 |  |
|  |  |  | Total 2 | 23.5()21-26) |  | yes |  |  |  |  |  |  |  |  |  |  | OHA 2 |  |
|  |  |  | Total 5 | 16.8(12-21) |  | yes |  |  |  |  |  |  |  |  |  |  | Lifestyles 3/OHA 2 | P379fsdelCT |
|  |  |  | Total 2 | 19(13-25) |  | yes |  |  |  |  |  |  |  |  |  |  | Lifestyles 1/INS 1 |  |
|  |  |  | Total 4 | 21.5(10-30) |  | yes |  |  |  |  |  |  |  |  |  |  | OHA 3/INS 1 | A443fsdekCA |
|  |  |  | Total 7 | 20.3(6-36) |  | yes |  |  |  |  |  |  |  |  |  |  | Lifestyles 1/OHA 2/INS 4 | P447L |
|  |  |  | Total 3 | 17(17-18) |  | yes |  |  |  |  |  |  |  |  |  |  | Lifestyles 2/INS 1 | P159L |
|  |  |  | Total 7 | 34(15,77) |  | yes |  |  |  |  |  |  |  |  |  |  | Lifestyles 1/OHA 5/INS 1 | T620I |
| François Godart, et al(77) | 2000 | The USA | male | 26 |  | yes |  |  |  |  |  |  |  |  |  |  | OHA | >35nt -218T>C |
|  |  |  | male | 19 |  | yes |  |  |  |  |  |  |  |  |  |  | OHA | >35NT -207-206ins27 |
|  |  |  | male | 20 |  | yes |  |  |  |  |  |  |  |  |  |  | Lifestyles | 15nt -187C>T |
|  |  |  | male | 4 |  | yes |  |  |  |  |  |  |  |  |  |  | Lifestyles | 12nt -119delG |
|  |  |  | female | 29 |  | yes |  |  |  |  |  |  |  |  |  |  | INS | 12nt -119G>A |
|  |  |  | female | 32 |  | yes |  |  |  |  |  |  |  |  |  |  | INS | -97T>G |
|  |  |  | male | 23 |  | yes |  |  |  |  |  |  |  |  |  |  | INS | -62C>G |
| Torben Hansen, et al(78) | 1997 | The USA | female | 16 | 21.2 | yes | 8.2 | 12.5 |  |  |  |  |  |  |  |  |  | Exon2 128 ATC(Ile)>AAC（Asn) |
|  |  |  | female | 13 | 21.4 | yes | 6.4 | 7.2 |  |  |  |  |  |  |  |  |  | Exon6 379 CCT(Pro)>CC (frameshift) |
|  |  |  | male | 18 | 22.1 | yes | 7.2 | 7.7 |  |  |  |  |  |  |  |  |  | Exon7 447 CCG(Pro)>CTG(Leu) |
|  |  |  | male | 7 | 21.5 | yes | 9.1 | 14.8 |  |  |  |  |  |  |  |  |  | Exon2 143 CAC(His)>TAC(Tyr) |
|  |  |  | male | 15 | 25.0 | yes | 9.2 | 12.1 |  |  |  |  |  |  |  |  |  |  |
|  |  |  | female | 12 | 33.7 | yes | 6.3 | 6.9 |  |  |  |  |  |  |  |  |  |  |
|  |  |  | female | 5 | 23.2 | yes | 6.0 | 7.0 |  |  |  |  |  |  |  |  |  |  |
|  |  |  | female | 17 | 40.2 | yes | 12.7 | 12.7 |  |  |  |  |  |  |  |  |  |  |
|  |  |  | female | 19 | 22.9 | yes | 6.1 | 6.1 |  |  |  |  |  |  |  |  |  | Exon9 559 GCA(Ala)>GCAA(frameshift) |
| Y. Horikawa, et al(79) | 2014 | japan | Male 6/female 18 | 11.9±2.9 | 19.5±2.8 | 22 patients |  | 7.1±1.2 |  | 1.5±0.5 |  |  |  |  |  |  | Lifestyles 4/OHA 10/INS 10 |  |
| T. Ikema, et al(80) | 2002 | japan | male | 1 |  |  |  |  |  |  |  |  |  |  |  | DR, DKD | INS | L518P519fsTCC>A+T521L+V617I |
|  |  |  | female | 47 | 23.4 |  |  |  |  |  |  |  |  |  |  |  | Lifestyles |  |
|  |  |  | male |  | 17.5 |  |  |  |  |  |  |  |  |  |  |  |  | L518P519fsTCC>A |
|  |  |  | female | 13 | 19.0 |  |  |  |  |  |  |  |  |  |  |  | INS | T512L+V617L |
|  |  |  | female | 10 | 20.9 |  |  |  |  |  |  |  |  |  |  |  | OHA | Primer A+102G>C |
|  |  |  | male | 38 | 23.5 |  |  |  |  |  |  |  |  |  |  |  | OHA |  |
| Timothy M, et al(81) | 1997 | England | Total 44 patients |  |  |  |  |  |  |  |  |  |  |  |  |  | Lifestyles 5/OHA 23/INS 10/OHA+INS 1 | P291fsinsC |
|  |  |  | Total 6 patients |  |  |  |  |  |  |  |  |  |  |  |  |  | OHA 5/INS 1 | P291fsdelC |
|  |  |  | Total 5 patients |  |  |  |  |  |  |  |  |  |  |  |  |  | Lifestyles 1/OHA 4 | W267X |
|  |  |  | Total 3 patients |  |  |  |  |  |  |  |  |  |  |  |  |  | Lifestyles 1/OHA 2 | E132K |
|  |  |  | Total 3 patients |  |  |  |  |  |  |  |  |  |  |  |  |  | OHA 1/INS 2 | IVS2nt+1G>A |
|  |  |  | Total 5 patients |  |  |  |  |  |  |  |  |  |  |  |  |  | INS 5 | IVS4nt-2A>G |
|  |  |  | Total 2 patients |  |  |  |  |  |  |  |  |  |  |  |  |  | INS 2 | D135fsdelA |
|  |  |  | Total 3 patients |  |  |  |  |  |  |  |  |  |  |  |  |  | Lifestyles 1/OHA 2 | A443fsdelCA |
|  |  |  | 1 patient | 29 |  |  |  |  |  |  |  |  |  |  |  |  | Lifestyles | Q474X |
|  |  |  | Total 2 patients |  |  |  |  |  |  |  |  |  |  |  |  |  | Lifestyles 2 | IVS8nt+1G>A |
|  |  |  | Total 3 patients |  |  |  |  |  |  |  |  |  |  |  |  |  | OHA 2/INS 1 | IVS9nt-1G>A |
| Maria Galán, et al(82) | 2010 | Spain | female | 20 | 21 | yes | 5.9 | 6.9 |  |  |  |  |  |  |  |  | Lifestyles | c.55G>C/exon1 delCACGCGG |
|  |  |  | male | 17 | 24 | yes | 7.4 | 10.2 |  |  |  |  |  |  |  |  | OHA | c.397G>A/p.Val133Met |
|  |  |  | female | 24 | 20 | yes | 9.6 | 7.9 |  | 1.9 |  |  |  |  |  |  | OHA | c.586A>G/p.Thr196Ala |
|  |  |  | female | 14 | 22 | yes | 10.6 | 14.2 |  | 1.0 |  |  |  |  |  |  | OHA+INS | c.704A>G/p.Glu235Gly |
|  |  |  | female | 17 | 22 | yes | 5.8 | 4.7 |  | 0.8 |  |  |  |  |  |  | OHA | c.811C>T/p.Arg271Trp |
|  |  |  | male | 34 | 25 | yes | 5.8 | 7.0 |  | 0.76 |  |  |  |  |  |  | Lifestyles | c.1136C>G/p.Pro379Arg |
| A. Johansen, et al(83) | 2005 | Denmark | Male 19/female 21 | 24.0±12.1 | 25.4±4.4 | 20 patients yes | 7.8±1.4 |  |  | 1.06±0.57 |  | 1.51±1.58 | 5.3±1.3 | 1.48±0.42 |  | DR 2/DKD 2/DN 1 | Lifestyles 2/OHA 7/INS 10 |  |
| K.-A. Kim, et al(84) | 2003 | Korea | male | 15 | 18.3 | yes |  | 5.1 | 18.8 | 0.30 | 0.99 |  |  |  |  |  | OHA+INS | R263L |
| Naoko Iwasaki, et al(85) | 1997 | The USA | male | 64 | 20.9 | yes |  | 6.2 |  |  |  |  |  |  |  | none | Lifestyles | G191D |
|  |  |  | male | 17 | 17.5 | yes |  | 5.1 |  |  |  |  |  |  |  | none | Lifestyles | K205Q |
|  |  |  | female | 15 | 19.5 | yes |  | 10.2 |  |  |  |  |  |  |  | none | OHA | P379fsdelCT |
|  |  |  | male | 13 | 20.3 | yes |  |  |  |  |  |  |  |  |  | DR, DKD | INS | L584S585fsinsTC |
|  |  |  | male | 28 | 22.2 | yes |  | 7.4 |  |  |  |  |  |  |  | none | Lifestyles | R263C |
|  |  |  | female | 16 | 22.4 | yes |  |  |  |  |  |  |  |  |  | DR | INS | T392fsdelA |
|  |  |  | female | 9 | 20.9 | yes |  |  |  |  |  |  |  |  |  | DR, DKD | INS | R131Q |
|  |  |  | male | 9 | 20.7 | yes |  | 6.1 |  |  |  |  |  |  |  | none | Lifestyles | L12H |
| T-S. Jap, et al(86) | 2000 | china | male | 23 | 19.81 | yes | 7.4 |  |  |  |  | 1.02 | 4.19 |  |  | DR | OHA |  |
| Henian Cao, et al(87) | 2002 | Canada | female | 19 |  | yes |  |  |  |  |  |  |  |  |  |  | OHA | c.105delCA/1051delCA |
|  |  |  | female | 13 |  | yes |  |  |  |  |  |  |  |  |  |  | OHA |  |
|  |  |  | male | 12 |  | yes |  |  |  |  |  |  |  |  |  |  | Lifestyles |  |
|  |  |  | female | 16 |  | yes |  |  |  |  |  |  |  |  |  |  | INS | c.415G>A/R131Q |
|  |  |  | female | 13 |  | yes |  |  |  |  |  |  |  |  |  |  | OHA |  |
|  |  |  | male | 38 |  | yes |  |  |  |  |  |  |  |  |  |  | OHA | c.771C>T/Q250X |
|  |  |  | male | 23 |  | yes |  |  |  |  |  |  |  |  |  |  | Lifestyles |  |
|  |  |  | female | 15 |  | yes |  |  |  |  |  |  |  |  |  |  | OHA |  |
| Soo Heon Kwak, et al(88) | 2016 | Korea | female | 19 | 21.3 | yes | 6.1 | 6.67 | 0.9 |  |  |  |  |  |  |  | OHA | c.415C>G/p.Leu139Val |
| Doddabelavangala Mruthyunjaya M, et al(89) | 2017 | India | female | 22 | 30 | yes |  |  |  |  |  |  |  |  |  |  | INS | c.8C>G/Ser3Cys |
| E. Stern, et al(90) | 2007 | Israel | male | 13 |  | yes | 11 | 9.94 |  |  |  |  |  |  |  |  | INS | Arg131Gln |
|  |  |  | female | 14.5 |  | yes | 10.4 |  |  |  |  |  |  |  |  |  | INS | Arg159Trp |
|  |  |  | male | 16 |  | yes | 7.1 | 6.56 |  |  |  |  |  |  |  |  | Lifestyles | Ala174fsdelCGCAGCGTAAG |
|  |  |  | male | 18 |  | yes | 4.6 | 15.56 |  |  |  |  |  |  |  |  | INS | Arg271Gln |
|  |  |  | male | 15 |  | yes | 6.1 | 6.56 |  |  |  |  |  |  |  |  | Lifestyles | Pro447Leu |
|  |  |  | male | 13 |  | yes | 11.8 | 23.17 |  |  |  |  |  |  |  |  | Lifestyles | Pro291fsinsC |
| Yorifuji T, et al(91) | 2012 | japan | female | 15 |  |  |  | 9.17 |  |  |  |  |  |  |  |  |  | c.1043T>C/p.L348P |
|  |  |  | female | 12 |  |  |  | 6.88 |  |  |  |  |  |  |  |  |  | c.779C>T/p.T260M |
|  |  |  | male | 9 |  |  |  | 6.88 |  |  |  |  |  |  |  |  |  | c.391C>T/p.R131W |
|  |  |  | male | 11 |  |  |  | 9.16 |  |  |  |  |  |  |  |  |  | Exon 7-9 deletion |
|  |  |  | male | 8 |  |  |  | 4.66 |  |  |  |  |  |  |  |  |  | c.788G>A/p.R263H |
|  |  |  | male | 13 |  |  |  | 4.61 |  |  |  |  |  |  |  |  |  | c.872delC/p.P291fs |
|  |  |  | female | 13 |  |  |  | 7.6 |  |  |  |  |  |  |  |  |  | c.1181delC/p.P394fs |
|  |  |  | female | 11 |  |  |  | 8.49 |  |  |  |  |  |  |  |  |  | c.1054delT/p.S352fs |
|  |  |  | female | 11 |  |  |  | 9.27 |  |  |  |  |  |  |  |  |  | c.392G>A/p.R131Q |
|  |  |  | female | 13 |  |  |  | 5.44 |  |  |  |  |  |  |  |  |  | c.872-873insC/p.P291fs |
|  |  |  | male | 14 |  |  |  | 4.27 |  |  |  |  |  |  |  |  |  | c.598C>T/p.R200W |
| S. Tanaka, et al(92) | 2000 | japan |  | 27 | 21.3 | yes | 6.4 | 10.9 |  |  |  |  |  |  |  |  | Lifestyles | T539fsdelC |
|  |  |  |  | 32 | 21.4 | yes |  | 7.3 |  |  |  |  |  |  |  |  | Lifestyles |  |
|  |  |  |  | 27 | 18.5 | yes |  | 7.5 |  |  |  |  |  |  |  |  |  | P291fsinsC |
|  |  |  |  | 33 | 18.7 | yes |  | 8.4 |  |  |  |  |  |  |  |  |  |  |
| Dalia Toaima, et al(93) | 2005 | Germany |  | 14 | 22.8 | yes | 6.8 | 8.0 |  | 0.97 |  |  |  |  |  |  | OHA | c.73G>C/A25P |
|  |  |  |  | 10 | 25.8 | yes | 7.1 | 8.8 |  | 2.3 |  |  |  |  |  |  | OHA | c.499G>A/R200Q |
|  |  |  |  | 15 | 21.7 | yes | 6.3 | 8.2 |  |  |  |  |  |  |  |  | INS | c.873-874insC/P291fsinsC |
|  |  |  |  | 16 | 22.0 | yes | 6.4 | 9.4 |  | 0.79 |  |  |  |  |  |  | INS | c.1155-1156insA/L383fsinsA |
|  |  |  |  | 11 | 20.2 | yes | 8.7 | 13.1 |  | 0.5 |  |  |  |  |  |  | INS | c.1323delG/R442fsX456 |
| Maurizio Delvecchio, et al(94) | 2014 | Italy | Children 6 | 14.1-17.8 |  |  | 5.9-8.1 | 6.06-8.28 |  |  |  |  |  |  |  |  | Lifestyles 3/OHA 1/INS 2 |  |
|  |  |  | Adults 3 | 17.9-19.6 |  |  | 7,2-9.5 | 6.89-12.17 |  |  |  |  |  |  |  |  | Lifestyles 1/INS 2 |  |
| Christina Tatsia,et al(95) | 2013 | Greece |  | 18 |  |  |  | 8.33 |  |  |  |  |  |  |  |  |  | C.319C>T/R131W |
|  |  |  |  | 14 |  |  |  | 8.89 |  |  |  |  |  |  |  |  |  | c.481G>A/A161T |
|  |  |  |  | 14 |  |  |  | 12.22 |  |  |  |  |  |  |  |  |  | c.481G>C/A161P |
|  |  |  |  | 8 |  |  |  | 8.5 |  |  |  |  |  |  |  |  |  | c.493T>C/W165R |
|  |  |  |  | 0.3 |  |  |  | 14.2 |  |  |  |  |  |  |  |  |  | c.599G>A/R200Q |
|  |  |  |  | 17 |  |  |  | 9.44 |  |  |  |  |  |  |  |  |  | c.607C>T/R203C |
|  |  |  |  | 16 |  |  |  |  |  |  |  |  |  |  |  |  |  | c.682-684delGAG/E228del |
|  |  |  |  | 8 |  |  |  | 16.67 |  |  |  |  |  |  |  |  |  | c.685C>T/R229X |
|  |  |  |  | 13.5 |  |  |  | 14.11 |  |  |  |  |  |  |  |  |  |  |
|  |  |  |  | 24 |  |  |  | 5.56 |  |  |  |  |  |  |  |  |  | c.788G>A/R263H |
|  |  |  |  | 20 |  |  |  | 8.17 |  |  |  |  |  |  |  |  |  | c.824-826delAAG/E275del |
|  |  |  |  | 18 |  |  |  | 11.11 |  |  |  |  |  |  |  |  |  | c.872dupC/G292fs |
|  |  |  |  | 23 |  |  |  | 6.94 |  |  |  |  |  |  |  |  |  | c.1177delT/S393fs |
|  |  |  |  | 13 |  |  |  | 11.61 |  |  |  |  |  |  |  |  |  | c.1201C>T/Q401X |
|  |  |  |  | 3 |  |  |  | 10.5 |  |  |  |  |  |  |  |  |  | c.1331-1332delAG/S445fs |
|  |  |  |  | 20 |  |  |  | 9.11 |  |  |  |  |  |  |  |  |  | c.1501G>A/A501T |
| BEATE KARGES, et al(96) | 2007 | Germany | female | 13 | 25 | yes | 8.5 | 9.3 | 13.4 | 0.9 |  |  |  |  |  |  | OHA | c.526+1delGTAA |
|  |  |  | female | 14 |  | yes | 6.0 | 7.3 | 10.1 | 1,8 |  |  |  |  |  |  |  |  |
|  |  |  | female | 19 |  | yes | 6.1 | 6.8 | 13.1 | 1.7 |  |  |  |  |  |  |  |  |
| Atsushi Iwabuchi, et al(97) | 2013 | japan | male | 1.6 | 15.3 | yes | 7.9 | 5.1 | 19.78 | 0.2 |  |  |  |  |  |  | INS | c.593delA/p.Lys198fs |
| H. U. Irgens, et al(98) | 2013 | Norway |  | 9 | 28.3 | yes | 7.2 |  |  |  |  |  |  |  |  |  | Lifestyles | c.686G>A/p.Arg229Gln |
|  |  |  |  | 9 | 19.4 | yes | 6.8 |  |  |  |  |  |  |  |  |  | OHA | c.1136-1137delCT/p.Pro379fs |
|  |  |  |  | 2 | 17.6 | no | 4.7 |  |  | 1.5 |  |  |  |  |  |  | Lifestyles | c.872dupC/p.Gly292fs |
|  |  |  |  | 13 |  | no | 7.9 |  |  | 0.4 |  |  |  |  |  |  |  |  |
|  |  |  |  | 13 | 18.1 | no | 8.5 |  |  |  |  |  |  |  |  |  | INS | c.686G>A/p.Arg229Gln |
|  |  |  |  | 9 | 18.3 | no | 8.7 |  |  | 0.9 |  |  |  |  |  |  | OHA | c.391C>T/p.Arg131Trp |
|  |  |  |  | 10 | 25.3 | yes | 8.6 |  |  | 1.0 |  |  |  |  |  |  | Lifestyles | c.686G>A/p.Arg229Gln |
|  |  |  |  | 12 | 28.9 | yes | 7.4 |  |  | 1.0 |  |  |  |  |  |  | Lifestyles | c.1745A>G/p.His582Arg |
|  |  |  |  | 14 | 18.6 | yes | 6.9 |  |  | 1.5 |  |  |  |  |  |  | OHA | c.872dupC/p.Gly292fs |
|  |  |  |  | 7 | 22.8 | yes | 6.8 |  |  | 0.45 |  |  |  |  |  |  | Lifestyles | c.956-2A>G |
|  |  |  |  | 13 | 21.9 | no | 8.3 |  |  | 0.88 |  |  |  |  |  |  | Lifestyles | c.1351A>G/p.Ser451Gly |
|  |  |  |  | 13 | 23.5 | yes | 10.4 |  |  | 0.66 |  |  |  |  |  |  | INS | c.666-668delGAA/p.Lys222del |
|  |  |  |  | 13 | 22.6 | yes | 7.1 |  |  | 0.78 |  |  |  |  |  |  | Lifestyles |  |
|  |  |  |  | 4 | 16.6 | no |  |  |  |  |  |  |  |  |  |  |  | c.872dupC/p.Gly292fs |
|  |  |  |  | 7 | 22.1 | yes | 5.8 |  |  | 1.24 |  |  |  |  |  |  | Lifestyles |  |
| S. Buchbinder, et al(99) | 2010 | Germany |  | 43 | 26 |  |  |  |  |  |  |  |  |  |  | none | OHA+INS | c.121G>T/p.E41X |
|  |  |  |  | 29 | 28 |  |  |  |  |  |  |  |  |  |  | DR, DN | OHA+INS |  |
|  |  |  |  | 30 | 24 |  |  |  |  |  |  |  |  |  |  | none | OHA+INS |  |
|  |  |  |  | 35 | 26 |  |  |  |  |  |  |  |  |  |  | none | Lifestyles |  |
|  |  |  |  | 18 | 26 |  |  |  |  |  |  |  |  |  |  | none | OHA |  |
|  |  |  |  | 63 | 26 |  |  |  |  |  |  |  |  |  |  | none | OHA |  |
|  |  |  |  | 38 | 22 |  |  |  |  |  |  |  |  |  |  | none | OHA |  |
|  |  |  |  | 53 | 29 |  |  |  |  |  |  |  |  |  |  | none | OHA |  |
|  |  |  |  | 21 | 29 |  |  |  |  |  |  |  |  |  |  | none | INS |  |
|  |  |  |  | 34 | 28 |  |  |  |  |  |  |  |  |  |  | none | Lifestyles |  |
| Z. Bazalova, et al(100) | 2010 | Czech Republic | male | 24 | 23.8 | yes | 5.0 | 7 |  | 2.21 |  | 0.56 | 3.50 | 1.04 | 2.21 | none | Lifestyles | c.802T>C/p.Phe268Leu |
|  |  |  | male | 47 | 28.0 | yes | 5.6 | 7.5 |  | 1.97 |  | 0.95 | 6.06 | 1.70 | 3.93 | none | Lifestyles |  |
|  |  |  | male | 42 | 34 | yes | 5.3 | 6.5 |  | 1.96 |  | 1.03 | 4.49 | 0.98 |  | none | OHA |  |
|  |  |  | male | 27 | 24.7 | yes | 7.7 | 8 |  | 0.90 |  | 0.83 | 5.19 |  |  | DR, DN | INS | c.871C>T/p.Pro291Ser |
|  |  |  | male | 27 | 24.5 | yes | 3.9 | 8.5 |  | 1.11 |  | 2.06 | 4.69 | 1.38 | 2.55 | none | Lifestyles |  |
| R. BARRIO, et al(101) | 2002 | Spain | Male 2/female 3 | 14.4±2.7 |  |  | 6.7±1.5 | 8.09±1.91 | 15.95±2.43 |  |  |  |  |  |  | DKD 3 | Lifestyles 1/OHA 2/INS 2 |  |
| G. Alkorta-Aranburu, et al(102) | 2014 | The USA | male | 10 |  |  |  |  |  |  |  |  |  |  |  |  | INS | c.391C>T/p.Arg131Trp |
|  |  |  | male | 3 |  |  |  |  |  |  |  |  |  |  |  |  | Lifestyles | c.586A>G/p.Thr196Ala |
| Suwattanee Kooptiwut, et al(103) | 2009 | Thailand | male | 12 | 20/41 | yes |  | 13.2 |  |  |  | 0.43 | 3.55 |  | 1.77 |  | OHA | G554fsX556 |
|  |  |  | female | 50 | 22.51 | yes |  | 11.71 |  |  |  | 0.723 | 4.82 |  | 3.24 | DR, DN | OHA |  |
|  |  |  | male | 52 | 18.34 | yes |  | 6.33 |  |  |  |  |  |  |  |  |  |  |
| Bente B. Johansson,et al(104) | 2016 | Norway |  | 6 | 15.5 | no | 10.8 |  |  |  |  |  |  |  |  |  | INS | c.66C>G/p.Ser22Arg |
|  |  |  |  | 14 | 15.8 | no | 11.4 |  |  |  |  |  |  |  |  |  | INS | c.1061C>T/p.Thr354Met |
|  |  |  |  | 13 | 24.4 | no | 6.2 |  |  |  |  |  |  |  |  |  | Lifestyles | c.335C>T/p.PRO112Leu |
|  |  |  |  | 13 | 24.1 | no | 6.6 |  |  |  |  |  |  |  |  |  |  |  |
|  |  |  |  | 9 | 18.3 | no | 8.7 |  |  |  |  |  |  |  |  |  | OHA | c.391C>T/p.Arg131Trp |
|  |  |  |  | 10 |  | yes | 10.6 |  |  |  |  |  |  |  |  |  | INS | c.523C>G/p.Gln175Glu |
|  |  |  |  | 14 | 26.3 | no |  |  |  |  |  |  |  |  |  |  | Lifestyles | c.608G>A/p.Arg203His |
|  |  |  |  | 13 | 27.8 | no |  |  |  |  |  |  |  |  |  |  | Lifestyles |  |
|  |  |  |  | 13 | 23.5 | yes | 10.4 |  |  |  |  |  |  |  |  |  | INS | c.666-668del/p.Lys222del |
|  |  |  |  | 13 | 22.6 | yes | 7.1 |  |  |  |  |  |  |  |  |  | Lifestyles |  |
|  |  |  |  | 10 | 25.3 | yes | 8.6 |  |  |  |  |  |  |  |  |  | Lifestyles | c.686G>A/p.Arg229Gln |
|  |  |  |  | 15 | 27.3 | yes | 7.0 |  |  |  |  |  |  |  |  |  |  |  |
|  |  |  |  | 7 | 22.1 | yes | 5.8 |  |  |  |  |  |  |  |  |  | Lifestyles | c.872dup/p.Gly292ArgfsTer25 |
|  |  |  |  | 2 | 17.6 | no | 4.7 |  |  |  |  |  |  |  |  |  | Lifestyles |  |
|  |  |  |  | 6 |  | no | 9.2 |  |  |  |  |  |  |  |  |  | INS | c.917G>T/p,Gly306Val |
|  |  |  |  | 12 |  | no |  |  |  |  |  |  |  |  |  |  |  | c.1061C>T/p.Thr354Met |
|  |  |  |  | 13 | 21.9 | no | 8.3 |  |  |  |  |  |  |  |  |  | Lifestyles | c.1351A>G/p.Ser451Gly |
|  |  |  |  | 11 | 27.1 | no | 7.0 |  |  |  |  |  |  |  |  |  | INS | c.1640-1641del/p.Thr547ArgfsTer5 |
|  |  |  |  | 8 | 14.2 | no | 9.7 |  |  |  |  |  |  |  |  |  | Lifestyles | c.1739C>T/p.Pro580Leu |
|  |  |  |  | 12 | 28.9 | yes | 7.4 |  |  |  |  |  |  |  |  |  | Lifestyles | c.1745A>G/p.His582Arg |
| Alessia Cappelli, et al(105) | 2009 | Italy | female | 17 |  |  |  |  |  |  |  |  |  |  |  |  | OHA | R159W |
|  |  |  | male | 13 |  |  |  |  |  |  |  |  |  |  |  |  | OHA | R272H |
|  |  |  | female | 16 |  |  |  |  |  |  |  |  |  |  |  |  | OHA+INS |  |
|  |  |  | female | 9 |  |  |  |  |  |  |  |  |  |  |  |  | OHA |  |
|  |  |  | female | 23 |  |  |  |  |  |  |  |  |  |  |  |  | INS |  |
|  |  |  | male | 16 |  |  |  |  |  |  |  |  |  |  |  |  | OHA | IVS4nt-1G>T |
|  |  |  | male | 30 |  |  |  |  |  |  |  |  |  |  |  |  | OHA |  |
| A Costa, et al(106) | 2000 | Spain | Ale 7/female 12 | 20.5±6.4 | 23.1±3.4 |  | 7.9±2.4 |  |  |  |  | 1.0±0.4 | 4.9±0.8 |  |  |  | Lifestyles 5.OHA 5/INS 9 |  |
| Klara Rozenkova, et al(107) | 2015 | Czech Republic | male | 6 |  | yes |  |  |  |  |  |  |  |  |  |  | OHA | p.Gly31Asp |
|  |  |  | male | 2.9 |  | yes |  |  |  |  |  |  |  |  |  |  | Lifestyles | p.Asn62Lysfs93* |
|  |  |  | male | 2.1 |  | yes |  |  |  |  |  |  |  |  |  |  | Lifestyles | p.Leu254Gln |
|  |  |  | male | 46.3 |  | yes |  |  |  |  |  |  |  |  |  |  | Lifestyles | p.Arg272His |
|  |  |  | female | 4.0 |  | yes |  |  |  |  |  |  |  |  |  |  | OHA | p.Glu508Lys |
| M. Lehto, et al(108) | 1999 | Sweden | Male 5/female 4 | 24±10 | 23.1±3.4 |  | 7.2±1.2 | 8.5±2.7 |  | 0.22±0.22 |  | 1.35±0.47 | 5.72±1.27 | 1.41±0.36 |  |  | Lifestyles 1/OHA 4/INS 4 |  |
| LISE BJØRKHAUG, et al(109) | 2003 | Norway | 1 | 12 |  |  |  |  |  |  |  |  |  |  |  |  | Lifestyles | G47E |
|  |  |  | 7 | 14-31 |  |  |  |  |  |  |  |  |  |  |  |  | Lifestyles 2/INS 1 | P112L |
|  |  |  | 11 | 10-20 |  |  |  |  |  |  |  |  |  |  |  | DR 1 | Lifestyles 1/OHA 4/INS 5 | R131W |
|  |  |  | 6 | 11-26 |  |  |  |  |  |  |  |  |  |  |  | DR 2/DN 1 |  | R171X |
|  |  |  | 1 | 31 |  |  |  |  |  |  |  |  |  |  |  |  | OHA | T196fsdelCCAA |
|  |  |  | 4 | 21-36 |  |  |  |  |  |  |  |  |  |  |  | DKD 1 | INS 3 | R229Q |
|  |  |  | 1 | 24 |  |  |  |  |  |  |  |  |  |  |  |  | OHA | IVS3-G>A |
|  |  |  | 1 |  |  |  |  |  |  |  |  |  |  |  |  |  |  | S256T |
|  |  |  | 6 | 13-27 |  |  |  |  |  |  |  |  |  |  |  |  |  | R263C |
|  |  |  | 1 | 16 |  |  |  |  |  |  |  |  |  |  |  |  | OHA | R271W |
|  |  |  | 1 | 24 |  |  |  |  |  |  |  |  |  |  |  | DKD 1 | Lifestyles | A276D |
|  |  |  | 31 | 6-54 |  |  |  |  |  |  |  |  |  |  |  | DR 5/DKD 1/DN 3 | Lifestyles 4/OHA 10/INS 14 | P291fsinsC |
|  |  |  | 2 | 11-20 |  |  |  |  |  |  |  |  |  |  |  |  | Lifestyles 1/OHA 1 | P379fsdelCT |
|  |  |  | 3 | 12-13 |  |  |  |  |  |  |  |  |  |  |  | DR | INS 2 | S445fsdelAG |
|  |  |  | 1 | 17 |  |  |  |  |  |  |  |  |  |  |  |  | Lifestyles 1 | P447L |
|  |  |  | 1 |  |  |  |  |  |  |  |  |  |  |  |  |  |  | Q446X |
|  |  |  | 1 | 35 |  |  |  |  |  |  |  |  |  |  |  |  | OHA 1/INS 1 | S531T |

Abbreviations: BMI, body mass index; HbA1c, glycated hemoglobin; FPG, fasting plasma glucose; PPG, post-prandial plasma glucose; FC, fasting C-peptide; PC, post-prandial C-peptide; TG, triglyceride; TC, total cholesterol; HDL-c, high-density lipoprotein cholesterol; LDL-c, low-density lipoprotein cholesterol; DR, diabetic retinopathy; DKD,diabetic kidney disease; DN, diabetic neuropathy; OHA, oral hypoglycemic drugs; INS, insulin.

**Supplementary Table 2.** Characteristics of patients with *HNF1-alpha* MODY in different types of *HNF1-alpha* mutations.

| Subject | Total (n = 492) | Missense mutations (n = 227) | Frameshift mutations (n = 198) | Nonsense mutations (n = 30) | Synonym mutations (n = 2) | Non-coding mutations (n=35) | P value |
| --- | --- | --- | --- | --- | --- | --- | --- |
| Age of diagnosis (years) | 20.1 (18.4,21.8) | 20.2 (17.3,23.1) | 20.2 (17.3,23.1) | 19.1 (16.8,21.5) | 19.5 (10.5,28.5) | 21.6 (17.7,25.5) | 0.881 |
| BMI (kg/m^2^) | 22.5 (20.3,24.7) | 23.3 (22.6,23.9) | 21.0 (20.3,21.6) | 25.8 (23.7,27.8) | NA | 20.5 (19.5,21.6) | < 0.001* |
| HbA1c (%) | 7.9 (7.1,8.8) | 8.2 (7.9,8.5) | 7.3 (6.9,7.7) | 7.2 (5.9,8.5) | NA | 9.5 (8.0,11.0) | < 0.001* |
| FPG (mmol/L) | 9.0 (8.5,9.6) | 9.3 (8.7,10.0) | 8.7 (8.2,9.2) | 8.6 (6.6,10.6) | NA | 10.1 (8.1,12.1) | 0.320 |
| 2h PG (mmol/L) | 17.5 (14.6,20.4) | 15.6 (14.7,16.4) | 14.5 (13.7,15.2) | 19.2 (18.2,20.3) | NA | 20.7 (19.7,21.8) | < 0.001* |
| Fasting C-peptide (ng/mL) | 0.87 (0.55,1.19) | 1.17 (1.08,1.26) | 0.98 (0.83,1.13) | 0.89 (0.62,1.16) | NA | 0.43 (0.33,0.53) | < 0.001* |
| 2-hour post-load C-peptide (ng/mL) | 2.42 (0.93,3.91) | 3.18 (2.91,3.45) | 1.66 (1.44,1.88) | NA | NA | NA | < 0.001* |
| TG (mmol/L) | 1.50 (1.37,1.64) | 1.51 (1.36,1.66) | 1.45 (1.07,1.83) | NA | NA | NA | 0.772 |
| TC (mmol/L) | 4.81 (4.31,5.30) | 5.03 (4.82,5.24) | 4.52 (4.10,4.94) | NA | NA | NA | 0.034* |
| HDL-c (mmol/L) | 1.23 (0.96,1.49) | 1.36 (1.25,1.47) | 1.09 (0.98,1.20) | NA | NA | NA | < 0.001* |
| LDL-c (mmol/L) | 3.08 (2.84,3.33) | 3.18 (2.95,3.41) | 2.92 (2.57,3.27) | NA | NA | NA | 0.226 |
| Male (%) | 46.7 (39.9,53.7) | 49.6 (40.9,58.4) | 32.5 (19.9,48.3) | 50.0 (26.0,74.0) | 100 (0,100) | 50.0 (29.4,70.6) | 0.448 |
| Family history (%) | 46.9 (27.2,67.6) | 38.7 (13.9,71.2) | 33.3 (14.9,58.9) | 100 (0,100) | 100 (0,100) | 94.4 (69.4,99.2) | 0.048* |
| Microvascular complications (%) | 40.7 (23.2,61.0) | 23.9 (15.2,35.5) | 45.5 (22.3,70.8) | 50.0 (16.8,83.2) | 50.0 (5.9,94.1) | 100 (0,100) | 0.389 |
| Diabetic retinopathy (%) | 33.3 (16.7,55.4) | 19.1 (11.2,30.6) | 31.1 (13.4,56.9) | 33.3 (8.4,73.2) | 50.0 (5.9,94.1) | 100 (0,100) | 0.699 |
| Diabetic kidney disease (%) | 17.7 (5.9,42.3) | 14.3 (7.3,26.1) | 9.8 (2.2,34.0) | NA | 0 (0,100) | 80.0 (30.9,97.3) | 0.046* |
| Diabetic neuropathy (%) | 20.0 (10.2,35.5) | 13.5 (6.6,25.7) | 23.4 (6.3,58.2) | 16.7 (2.3,63.1) | 50.0 (5.9,94.1) | 20.0 (2.7,69.1) | 0.733 |
| Lifestyles (%) | 22.7 (18.7,27.2) | 22.9 (17.2,29.7) | 25.2 (15.9,37.4) | 15.0 (4.9,37.6) | 0 (0,100) | 52.3 (2.4,98.0) | 0.858 |
| OHA (%) | 43.0 (38.2,48.0) | 43.5 (36.3,51.1) | 44.0 (36.1,52.3) | 47.5 (14.0,83.4) | 100 (0,100) | 34.6 (19.1,54.3) | 0.931 |
| INS (%) | 37.0 (29.4,45.3) | 29.3 (23.1,36.3) | 34.3 (25.8,44.1) | 26.7 (10.4,53.3) | 0 (0,100) | 59.1 (22.8,87.6) | 0.568 |

Abbreviations: BMI, body mass index; HbA1c, glycated hemoglobin; FPG, fasting plasma glucose; 2-hour PG, 2-hour post-load glucose; TG, triglyceride; TC, total cholesterol; HDL-c, high-density lipoprotein cholesterol; LDL-c, low-density lipoprotein cholesterol; OHA, oral hypoglycemic drugs; INS, insulin.

# References

1. Xiaoyan Ren, Min Liu, Chaoli Yan, et al. HNF-1α gene mutation in a family with MODY3 diabetes mellitus and literature review. Chin J Diffic and Compl Cas. 2021;20(8):838-40. doi: 10.3969/j.issn.1671-6450.2021.08.018.

2. Mengruo Xue. Clinical screening and analysis of early-onset diabetes and molecular genetic study of 2 patients with MODY3. Inner mongolia medical university. 2018.

3. Xiaoli Kang, Wenyu Ding, Xing Chen, et al. Clinical characteristics and HNF1ɑ gene analysis of a family with early-onset diabetes mellitus. Jouranl of prectical diabetology. 2017;20(5):5-6,57.

4. Desiatkina L. A family study and genetic screening of the suspected MODY3 (HNF 1 alpha) adolescent[D]. Dalian Medical University. 2015.

5. Wang Tianping, Zhang Miao, Shi Lixin, et al. A novel hepatocyte nuclear factor-1α genetic mutation in a Chinese pedigree with maturity-onset diabetes of the young type. Chin J Diabetes Mellitus. 2014(1):27-31. doi:10.3760/cma.j.issn.1674-5809.2014.01.006.

6. Pan Xin, Wang Xiaoli, Wang qiuyue, et al. Clinical characteristics of a family with maturity onset diabetes of the young type 3. Chin J Diabetes Mellitus. 2021;13(5):498-500. doi: 10.3760/cma.j.cn115791-20201203-00702.

7. Yuecheng Zhang, Liu Li, Xianyuan Luo, et al. Gene mutation analysis of a case of type 3 "juvenile-onset adult-onset diabetes mellitus". China Medical Engineering. 2015;23(9):2.

8. Yipaerguli. Ainiwaner. Genetic screening and pedigree analysis of clinically suspected MODY[D]. Xinjiang Medical University. 2021.

9. Shu Yanwen, Gu Tianwei, Shen Shanmei, et al. Three cases of maturity onset diabetes of the young and literature review. Int J Endocrinol Metab. 2021;41(03):237-40. doi: 10.3760/cma.j.cn121383-2020079-07076.

10. Shao Mingwei, Liu Yanling, Du Peijie, et al. Maturity-onset diabetes of the young type 3 caused by genetic mutation of hepatocyte nuclear factor-1a: one family report. Chin J Endocrinol Metab. 2020;36(7):603-6. doi: 10.3760/cma.j.cn311282-20200302-00118.

11. Ying Zhang, Yuan Jiang, Wenjuan Li, et al. A case of adult-onset diabetes type 3 in an adolescent. J Clin Inter Med. 2021;38(10):701-3. doi: 10.3969/j.issn.1001-9057.2021.10.017.

12. Pace NP, Rizzo C, Abela A, Gruppetta M, Fava S, Felice A, et al. Identification of an HNF1A p.Gly292fs Frameshift Mutation Presenting as Diabetes During Pregnancy in a Maltese Family. Clin Med Insights Case Rep. 2019;12:1179547619831034. doi: 10.1177/1179547619831034.

13. Pinés Corrales PJ, López Garrido MP, Louhibi Rubio L, Aznar Rodríguez S, López Jiménez LM, Lamas Oliveira C, et al. Importance of clinical variables in the diagnosis of MODY2 and MODY3. Endocrinol Nutr. 2011;58(7):341-6. doi: 10.1016/j.endonu.2011.05.002.

14. Giuffrida FMA, Moises RS, Weinert LS, Calliari LE, Manna TD, Dotto RP, et al. Maturity-onset diabetes of the young (MODY) in Brazil: Establishment of a national registry and appraisal of available genetic and clinical data. Diabetes Res Clin Pract. 2017;123:134-42. doi: 10.1016/j.diabres.2016.10.017.

15. Jesić MD, Sajić S, Jesić MM, Maringa M, Micić D, Necić S. A case of new mutation in maturity-onset diabetes of the young type 3 (MODY 3) responsive to a low dose of sulphonylurea. Diabetes Res Clin Pract. 2008;81(1):e1-3. doi: 10.1016/j.diabres.2008.03.005.

16. Fu J, Wang T, Zhai X, Xiao X. Primary hepatocellular adenoma due to biallelic HNF1A mutations and its co-occurrence with MODY 3: case-report and review of the literature. Endocrine. 2020;67(3):544-51. doi: 10.1007/s13300-019-0647-x.

17. N. T, Y. T, K. O, N. K, Y. H, M. M, et al. High frequency of mutations in the HNF-1alpha gene in non-obese patients with diabetes of youth in Japanese and identification of a case of digenic inheritance. Diabetologia. 2002;45(12):1709-12. oi: 10.1007/s00125-002-0978-3.

18. Awa WL, Thon A, Raile K, Grulich-Henn J, Meissner T, Schober E, et al. Genetic and clinical characteristics of patients with HNF1A gene variations from the German-Austrian DPV database. Eur J Endocrinol. 2011;164(4):513-20. doi: 10.1530/EJE-10-0842.

19. Knebel B, Mack S, Haas J, Herman-Friede MK, Lange S, Schubert O, et al. Divergent phenotypes in siblings with identical novel mutations in the HNF-1α gene leading to maturity onset diabetes of the young type 3. BMC Medical Genetics. 2016;17(1). doi: 10.1186/s12881-016-0297-z.

20. Fehmann HC, Groß U, Epe M. A New Mutation in the Hepatocyte Nuclear Factor-1-Alpha Gene (P224S) in a Newly Discovered German Family with Maturity-Onset Diabetes of the Young 3 (MODY 3). Family Members Carry Additionally the Homozygous 127L Amino Acid Polymorphism in the HNF1 Alpha Gene. Experimental and Clinical Endocrinology and Diabetes. 2004;112(2):84-7. doi: 10.1055/s-2004-815755.

21. Bacon S, Kyithar MP, Rizvi SR, Donnelly E, McCarthy A, Burke M, et al. Successful maintenance on sulphonylurea therapy and low diabetes complication rates in a HNF1A-MODY cohort. Diabet Med. 2016;33(7):976-84. doi: 10.1111/dme.12992.

22. Egan AM, Cunningham A, Jafar-Mohammadi B, Dunne FP. Diabetic ketoacidosis in the setting of HNF1A-maturity onset diabetes of the young. BMJ Case Rep. 2015;2015. doi: 10.1136/bcr-2014-209163.

23. Besser REJ, Jones J, McDonald TJ, Smith R, Shepherd MH, Hattersley AT. Using highly sensitive C-reactive protein measurement to diagnose MODY in a family with suspected type 2 diabetes. BMJ Case Reports. 2012. doi: 10.1136/bcr.01.2012.5612.

24. Misra S, Hassanali N, Bennett AJ, Juszczak A, Caswell R, Colclough K, et al. Homozygous Hypomorphic HNF1A Alleles Are a Novel Cause of Young-Onset Diabetes and Result in Sulfonylurea-Sensitive Diabetes. Diabetes care. 2020;43(4):909-12. doi: 10.2337/dc19-1843.

25. Docena MK, Faiman C, Stanley CM, Pantalone KM. Mody-3: novel HNF1A mutation and the utility of glucagon-like peptide (GLP)-1 receptor agonist therapy. Endocr Pract. 2014;20(2):107-11. doi: 10.4158/EP13254.OR.

26. Lebenthal Y, Fisch Shvalb N, Gozlan Y, Tenenbaum A, Tenenbaum-Rakover Y, Vaillant E, et al. The unique clinical spectrum of maturity onset diabetes of the young type 3. Diabetes Res Clin Pract. 2018;135:18-22. doi: 10.1016/j.diabres.2017.10.024.

27. Khelifa SB, Barboura l, Dandana A, Ferchichi S, Miled A. Le diabète de type MODY : revue générale et récentes découvertes. Annales de biologie clinique. 2011;69(5):531-40. doi: 10.1016/j.diabres.2016.01.015.

28. Szopa M, Kapusta M, Matejko B, Klupa T, Koblik T, Kiec-Wilk B, et al. Comparison of Glomerular Filtration Rate Estimation from Serum Creatinine and Cystatin C in HNF1A-MODY and Other Types of Diabetes. Journal of Diabetes Research, Vol 2015 (2015). 2015. doi: 10.1055/s-0035-1559605.

29. Iwen KA, Klein J, Hubold C, Lehnert H, Weitzel JM. Maturity-onset diabetes of the young and hepatic adenomatosis - characterisation of a new mutation. Exp Clin Endocrinol Diabetes. 2013;121(6):368-71. doi: 10.1055/s-0033-1341519.

30. Iwabuchi A, Kamoda T, Shinohara H, Sumazaki R. Japanese boy with maturity-onset diabetes of the young type 3 who developed diabetes at 19 months old. Pediatr Int. 2013;55(2):e32-4. doi: 10.1111/j.1442-200X.2012.03741.x.

31. Isomaa B, Henricsson M, Lehto M, Forsblom C, Karanko S, Sarelin L, et al. Chronic diabetic complications in patients with MODY3 diabetes. Diabetologia. 1998;41(4):467-73. doi: 10.1007/s001250050931.

32. Hummel M, Vasseur F, Mathieu C, Bellanne-Chantelot C, Froguel P, Standl E, et al. Two Caucasian families with the hepatocyte nuclear factor-1alpha mutation Tyr218Cys. Exp Clin Endocrinol Diabetes. 2007;115(1):62-4. doi: 10.1055/s-2007-955099.

33. Estalella I, Rica I, Perez de Nanclares G, Bilbao JR, Vazquez JA, San Pedro JI, et al. Mutations in GCK and HNF-1alpha explain the majority of cases with clinical diagnosis of MODY in Spain. Clin Endocrinol (Oxf). 2007;67(4):538-46. doi: 10.1111/j.1365-2265.2007.02921.x.

34. Bonatto N, Nogaroto V, Svidnicki PV, Milléo FQ, Grassiolli S, Almeida MC, et al. Variants of the HNF1α gene: A molecular approach concerning diabetic patients from southern Brazil. Genet Mol Biol. 2012;35(4):737-40. doi: 10.1590/S1415-47572012005000061.

35. Fendler W, Borowiec M, Antosik K, Szadkowska A, Deja G, Jarosz-Chobot P, et al. HDL cholesterol as a diagnostic tool for clinical differentiation of GCK-MODY from HNF1A-MODY and type 1 diabetes in children and young adults. Clin Endocrinol (Oxf). 2011;75(3):321-7. doi: 10.1111/j.1365-2265.2011.04052.x.

36. Fang C, Huang J, Huang Y, Chen L, Chen X, Hu J. A novel nonsense mutation of the HNF1α in maturity-onset diabetes of the young type 3 in Asian population. Diabetes Research and Clinical Practice. 2015;109(2):e5-e7. doi: 10.1016/j.diabres.2015.05.026.

37. Plengvidhya N, Tangjittipokin W, Teerawattanapong N, Narkdontri T, Yenchitsomanus PT. HNF1A mutation in a Thai patient with maturity-onset diabetes of the young: A case report. World J Diabetes. 2019;10(7):414-20. doi: 10.1111/j.1365-2265.2008.03397.x.

38. Weinert LS, Silveiro SP, Giuffrida FMA, Cunha VT, Bulcao C, Calliari LE, et al. Three unreported glucokinase (GCK) missense mutations detected in the screening of thirty-two Brazilian kindreds for GCK and HNF1A-MODY. Diabetes research and clinical practice. 2014;106(2):E44-E8. doi: 10.1016/j.diabres.2014.08.006.

39. Wang X, Wang T, Yu M, Zhang H, Ping F, Zhang Q, et al. Screening of HNF1A and HNF4A mutation and clinical phenotype analysis in a large cohort of Chinese patients with maturity-onset diabetes of the young. Acta Diabetol. 2019;56(3):281-8. doi: 10.1007/s00592-018-1232-x.

40. Tatsi EB, Kanaka-Gantenbein C, Scorilas A, Chrousos GP, Sertedaki A. Next generation sequencing targeted gene panel in Greek MODY patients increases diagnostic accuracy. Pediatr Diabetes. 2020;21(1):28-39. doi: 10.1111/pedi.12032.

41. Szopa M, Matejko B, Ucieklak D, Uchman A, Hohendorff J, Mrozińska S, et al. Quality of life assessment in patients with HNF1A-MODY and GCK-MODY. Endocrine. 2019;64(2):246-53. doi: 10.1055/s-0035-1559605.

42. Skupien J, Gorczynska-Kosiorz S, Klupa T, Cyganek K, Wanic K, Borowiec M, et al. Molecular background and clinical characteristics of HNF1A MODY in a Polish population. Diabetes Metab. 2008;34(5):524-8. doi: 10.1016/j.diabet.2008.05.004.

43. Rafique I, Saqib MAN, Mir A, Naeem M. Maturity Onset Diabetes of the Young – An Overview of Common Types. A Review. Romanian Journal of Diabetes Nutrition and Metabolic Diseases. 2018;25(2):209-13. doi: 10.1007/s13410-021-00926-8.

44. Miura J, Sanaka M, Ikeda Y, Watanabe C, Nakagami T, Iwasaki N, et al. A case of Type-1 diabetes mellitus formerly diagnosed as maturity-onset diabetes of the young (MODY) carrying suggestive MODY3 gene. Diabetes Research and Clinical Practice. 1997. doi: 10.1016/S0168-8227(97)00092-2.

45. Lopez AP, Foscaldi SA, Perez MS, Rodriguez M, Traversa M, Puchulu FM, et al. HNF1 alpha gene coding regions mutations screening, in a Caucasian population clinically characterized as MODY from Argentina. Diabetes Res Clin Pract. 2011;91(2):208-12. doi: 10.1016/j.diabres.2010.11.024.

46. McDonald TJ, McEneny J, Pearson ER, Thanabalasingham G, Szopa M, Shields BM, et al. Lipoprotein composition in HNF1A-MODY: Differentiating between HNF1A-MODY and Type 2 diabetes. Clinica chimica acta: International journal of clinical chemistry and applied molecular biology. 2012;413(9/10):927-32. doi: 10.1016/j.cca.2012.02.005.

47. Pruhova S, Dusatkova P, Neumann D, Hollay E, Cinek O, Lebl J, et al. Two Cases of Diabetic Ketoacidosis in HNF1A-MODY Linked to Severe Dehydration: Is it time to change the diagnostic criteria for MODY? Diabetes care. 2013;36(9):2573-4. doi: 10.1007/s00125-002-1010-7.

48. Tuomi T, Honkanen EH, Isomaa B, Sarelin L, Groop LC. Improved prandial glucose control with lower risk of hypoglycemia with nateglinide than with glibenclamide in patients with maturity-onset diabetes of the young type 3. Diabetes Care. 2006;29(2):189-94. doi: 10.2337/diacare.29.02.06.dc05-1314.

49. Ovsyannikova AK, Rymar OD, Ivanoshchuk DE, Mikhailova SV, Shakhtshneider EV, Orlov PS, et al. A Case of Maturity Onset Diabetes of the Young (MODY3) in a Family with a Novel HNF1A Gene Mutation in Five Generations. Springer Healthcare. 2018;9(1). doi: 10.1007/s13300-017-0350-8.

50. Mongolu S, Clarke P, Mansell P. It is never too late for sulfonylurea treatment in maturity onset diabetes of the young (MODY) Type 3? Diabetic Medicine. 2009;26:102.

51. Schnedl WJ, Holasek SJ, Schenk M, Enko D, Mangge H. Diagnosis of hepatic nuclear factor 1A monogenic diabetes mellitus (HNF1A-MODY) impacts antihyperglycemic treatment. Wien Klin Wochenschr. 2021;133(5-6):241-4. doi: 10.1007/s00508-020-01770-2.

52. Salzano G, Passanisi S, Mammì C, Priolo M, Pintomalli L, Caminiti L, et al. Maturity Onset Diabetes of the Young is Not Necessarily Associated with Autosomal Inheritance: Case Description of a De Novo HFN1A Mutation. Diabetes Ther. 2019;10(4):1543-8. doi: 10.1007/s13300-019-0633-3.

53. Becker M, Galler A, Raile K. Meglitinide analogues in adolescent patients with HNF1A-MODY (MODY 3). Pediatrics. 2014;133(3):e775-9. doi: 10.1542/peds.2012-2537.

54. Habeb AM, George ET, Mathew V, Hattersley AL. Response to oral gliclazide in a pre-pubertal child with hepatic nuclear factor-1 alpha maturity onset diabetes of the young. Ann Saudi Med. 2011;31(2):190-3. doi: 10.4103/0256-4947.75590.

55. Maltoni G, Zucchini S, Scipione M, Mantovani V, Salardi S, Cicognani A. Onset of type 1 diabetes mellitus in two patients with maturity onset diabetes of the young. Pediatric Diabetes. 2012;13(2):208-12. doi: 10.1111/j.1399-5448.2011.00788.x.

56. Nakamura A, Ishidu K, Tajima T. Early onset of liver steatosis in a Japanese girl with maturity-onset diabetes of the young type 3 (MODY3). J Clin Res Pediatr Endocrinol. 2012;4(2):104-6. doi: 10.4274/jcrpe.584.

57. Ng MCY, Li JKY, So WY, Critchley JAJH, Chan JCN. Nature or nurture: an insightful illustration from a Chinese family with hepatocyte nuclear factor-1 alpha diabetes (MODY3). Diabetologia. 2000;43(6):816. doi: 10.1007/s001250051382.

58. Quintos JB, Pingul MM, Boney CM. Arteriovascular calcification leading to diagnosis of maturity-onset diabetes of the young type 3. J Pediatr. 2013;163(2):608-.e1. doi: 10.1016/j.jpeds.2013.03.005.

59. Selwood M, Owen K. Keeping diabetes in the family: lessons from a family with HNF-1α MODY. European Diabetes Nursing. 2008;5(2):70-4. doi: 10.1002/edn.113.

60. Naoko I, Masashi T, Kuniya A, Wataru S, Atsushi W, Makiko O, et al. Pancreatic developmental defect evaluated by celiac artery angiography in a patient with MODY5. Human Genome Variation. 2016;3(1). doi: 10.1038/hgv.2016.22.

61. Kyithar MP, Bacon S, Pannu KK, Rizvi SR, Colclough K, Ellard S, et al. Identification of HNF1A-MODY and HNF4A-MODY in Irish families: phenotypic characteristics and therapeutic implications. Diabetes Metab. 2011;37(6):512-9. doi: 10.1016/j.diabet.2011.04.002.

62. Doria A, Yang Y, Malecki M, Scotti S, Dreyfus J, O'Keeffe C, et al. Phenotypic characteristics of early-onset autosomal-dominant type 2 diabetes unlinked to known maturity-onset diabetes of the young (MODY) genes. Diabetes Care. 1999;22(2):253-61. doi: 10.2337/diacare.22.2.253.

63. Zhang M, Wang T, Shi L, Yang Y. Hepatocyte nuclear factor‐α genetic mutation in a Chinese pedigree with maturity‐onset diabetes of the young (MODY3). Diabetes/Metabolism Research and Reviews. 2015;31(7):767-70. doi: 10.1002/dmrr.2678.

64. Plengvidhya N, Boonyasrisawat W, Chongjaroen N, Jungtrakoon P, Sriussadaporn S, Vannaseang S, et al. Mutations of maturity-onset diabetes of the young (MODY) genes in Thais with early-onset type 2 diabetes mellitus. Clin Endocrinol (Oxf). 2009;70(6):847-53. doi: 10.1111/j.1365-2265.2008.03397.x.

65. Pruhova S, Ek J, Lebl J, Sumnik Z, Saudek F, Andel M, et al. Genetic epidemiology of MODY in the Czech republic: new mutations in the MODY genes HNF-4alpha, GCK and HNF-1alpha. Diabetologia. 2003;46(2):291-5. doi: 10.1007/s00125-002-1010-7.

66. Stanik J, Dusatkova P, Cinek O, Valentinova L, Huckova M, Skopkova M, et al. De novo mutations of GCK, HNF1A and HNF4A may be more frequent in MODY than previously assumed. Diabetologia: Clinical and Experimental Diabetes and Metabolism = Organ of the European Association for the Study of Diabetes (EASD). 2014;57(3):480-4. doi: 10.1007/s00125-013-3119-2.

67. Malecki MT, Skupien J, Gorczynska-Kosiorz S, Klupa T, Nazim J, Moczulski DK, et al. Renal malformations may be linked to mutations in the hepatocyte nuclear factor-1alpha (MODY3) gene. Diabetes Care. 2005;28(11):2774-6. doi: 10.2337/diacare.28.11.2774.

68. Thanabalasingham G, Pal A, Selwood MP, Dudley C, Fisher K, Bingley PJ, et al. Systematic assessment of etiology in adults with a clinical diagnosis of young-onset type 2 diabetes is a successful strategy for identifying maturity-onset diabetes of the young. Diabetes Care. 2012;35(6):1206-12. doi: 10.2337/dc11-1243.

69. Maraschin JF, Kannengiesser C, Murussi N, Campagnolo N, Canani LH, Gross JL, et al. HNF1alpha mutations are present in half of clinically defined MODY patients in South-Brazilian individuals. Arquivos brasileiros de endocrinologia e metabologia. 2008;52(8):1326-31. doi: 10.1590/s0004-27302008000800020.

70. Pihoker C, Gilliam LK, Ellard S, Dabelea D, Davis C, Dolan LM, et al. Prevalence, characteristics and clinical diagnosis of maturity onset diabetes of the young due to mutations in HNF1A, HNF4A, and glucokinase: results from the SEARCH for Diabetes in Youth. J Clin Endocrinol Metab. 2013;98(10):4055-62. doi: 10.1210/jc.2013-1279.

71. Xu JY, Dan QH, Chan V, Wat NM, Tam S, Tiu SC, et al. Genetic and clinical characteristics of maturity-onset diabetes of the young in Chinese patients. Eur J Hum Genet. 2005;13(4):422-7. doi: 10.1038/sj.ejhg.5201347.

72. Yoshiuchi I, Yamagata K, Yang Q, Iwahashi H, Okita K, Yamamoto K, et al. Three new mutations in the hepatocyte nuclear factor-1alpha gene in Japanese subjects with diabetes mellitus: clinical features and functional characterization. Diabetologia. 1999;42(5):621-6. doi: 10.1007/s001250051204.

73. Cox RD, Southam L, Hashim Y, Horton V, Mehta Z, Taghavi J, et al. UKPDS 31: Hepatocyte nuclear factor-1alpha (the MODY3 gene) mutations in late onset Type II diabetic patients in the United Kingdom. United Kingdom prospective diabetes study. Diabetologia. 1999;42(1):120-1. doi: 10.1007/s001250051127.

74. Demol S, Lebenthal Y, Bar-Meisels M, Phillip M, Gat-Yablonski G, Gozlan Y. A family with a novel termination mutation in hepatic nuclear factor 1α in maturity-onset diabetes of the young type 3 which is unresponsive to sulphonylurea therapy. Hormone research in p?diatrics. 2014;81(4):280-4. doi: 10.1159/000356925.

75. Domínguez-López A, Miliar-García A, Segura-Kato YX, Riba L, Esparza-López R, Ramírez-Jiménez S, et al. Mutations in MODY genes are not common cause of early-onset type 2 diabetes in Mexican families. Jop. 2005;6(3):238-45. PMID: 15883474.

76. M.Frayling T, C.Evans J, P.Bulman M. β-Cell genes and diabetes molecular and clinical characterization of mutations in transcription factors. Diabetes: A Journal of the American Diabetes Association. 2001;50(Suppl.1):S94-S100. doi: 10.2337/diabetes.50.2007.s94.

77. Godart F, Bellanné-Chantelot C, Clauin S, Gragnoli C, Abderrahmani A, Blanché H, et al. Identification of seven novel nucleotide variants in the hepatocyte nuclear factor-1alpha (TCF1) promoter region in MODY patients. Hum Mutat. 2000;15(2):173-80. doi: 10.1002/(SICI)1098-1004(200002)15:2<173::AID-HUMU6>3.0.CO;2-W.

78. Hansen T, Eiberg H, Rouard M, Vaxillaire M, Moller AM, Rasmussen SK, et al. Novel MODY3 Mutations in the Hepatocyte Nuclear Factor-1  Gene: Evidence for a Hyperexcitability of Pancreatic  -cells to Intravenous Secretagogues in a Glucose-Tolerant Carrier of a P447L Mutation. Diabetes. 1997. doi: 10.2337/diab.46.4.726.

79. Horikawa Y, Enya M, Fushimi N, Fushimi Y, Takeda J. Screening of diabetes of youth for hepatocyte nuclear factor 1 mutations: clinical phenotype of HNF1β-related maturity-onset diabetes of the young and HNF1α-related maturity-onset diabetes of the young in Japanese. Diabetic Medicine. 2014;31(6):721-7. doi: 10.1111/dme.12416.

80. Ikema T, Shimajiri Y, Komiya I, Tawata M, Sunakawa S, Yogi H, et al. Identification of three new mutations of the HNF-1 alpha gene in Japanese MODY families. Diabetologia. 2002;45(12):1713-8. doi: 10.1007/s00125-002-0972-9.

81. Frayling TM, Bulman MP, Ellard S, Appleton M, Dronsfield MJ, Mackie AD, et al. Mutations in the hepatocyte nuclear factor–1α gene are a common cause of maturity-onset diabetes of the young in the UK. Diabetes. 1997;46(4):720-5. doi: 10.2337/diab.46.4.720.

82. Galán M, García-Herrero CM, Azriel S, Gargallo M, Durán M, Gorgojo JJ, et al. Differential effects of HNF-1α mutations associated with familial young-onset diabetes on target gene regulation. Mol Med. 2011;17(3-4):256-65. doi: 10.2119/molmed.2010.00097.

83. Johansen A, Jensen DP, Bergholdt R, Mortensen HB, Pociot F, Nerup J, et al. IRS1, KCNJ11, PPARγ2 and HNF-1α: Do amino acid polymorphisms in these candidate genes support a shared aetiology between type 1 and type 2 diabetes? Diabetes, Obesity and Metabolism. 2006;8(1):75-82. doi: 10.1210/jc.2005-0196.

84. Kim KA, Kang K, Chi YI, Chang I, Lee MK, Kim KW, et al. Identification and functional characterization of a novel mutation of hepatocyte nuclear factor-1α gene in a Korean family with MODY3. Diabetologia. 2003;46(5):721-7. doi: 10.1007/s00125-003-1079-7.

85. Iwasaki N, Oda N, Ogata M, Hara M, Bell GI. Mutations in the Hepatocyte Nuclear Factor-1α/MODY3 Gene in Japanese Subjects With Early- and Late-Onset NIDDM. Diabetes. 1997;46(9):1504. doi: 10.2337/diab.46.9.1504.

86. Jap TS, Wu YC, Chiou JY, Kwok CF. A novel mutation in the hepatocyte nuclear factor‐1α/MODY3 gene in Chinese subjects with early‐onset Type 2 diabetes mellitus in Taiwan. Diabetic Medicine. 2000;17(5):390-3. doi: 10.1046/j.1464-5491.2000.00285.x.

87. Cao H, Shorey S, Robinson J, Metzger DL, Stewart L, Cummings E, et al. GCK and HNF1A mutations in Canadian families with maturity onset diabetes of the young (MODY). Human Mutation. 2002;20(6):478-9. doi: 10.1002/humu.9090.

88. Kwak SH, Jung CH, Ahn CH, Park J, Chae J, Jung HS, et al. Clinical whole exome sequencing in early onset diabetes patients. Diabetes Res Clin Pract. 2016;122:71-7. doi: 10.1016/j.diabres.2016.10.005.

89. Doddabelavangala Mruthyunjaya M, Chapla A, Hesarghatta Shyamasunder A, Varghese D, Varshney M, Paul J, et al. Comprehensive Maturity Onset Diabetes of the Young (MODY) Gene Screening in Pregnant Women with Diabetes in India. PLoS One. 2017;12(1):e0168656. doi: 10.1371/journal.pone.0168656.

90. E. Stern, C. Strihan, O. Potievsky, R. Nimri, S. Shalitin, O. Cohen, et al. Four Novel Mutations, Including the First Gross Deletion in TCF1, identified in HNF4a, GCK and TCF1 in Patients with MODY in Israel. Journal of Pediatric Endocrinology and Metabolism. 2007;20(8):909-22. doi: 10.1515/jpem.2007.20.8.909.

91. Yorifuji T, Fujimaru R, Hosokawa Y, Tamagawa N, Shiozaki M, Aizu K, et al. Comprehensive molecular analysis of Japanese patients with pediatric‐onset MODY‐type diabetes mellitus. Pediatric Diabetes. 2012;13(1):26-32. doi: 10.1111/j.1399-5448.2011.00827.x.

92. Tanaka S, Kobayashi T, Tomura H, Okubo M, Nakanishi K, Takeda J, et al. A novel dominant-negative mutation of the hepatocyte nuclear factor-1alpha gene in Japanese early-onset type 2 diabetes. Horm Metab Res. 2000;32(9):373-7. doi: 10.1055/s-2007-978656.

93. Toaima D, Näke A, Wendenburg J, Praedicow K, Rohayem J, Engel K, et al. Identification of novel GCK and HNF1A/TCF1 mutations and polymorphisms in German families with maturity‐onset diabetes of the young (MODY). Human Mutation. 2005;25(5):503-4. doi: 10.1002/humu.9334.

94. Delvecchio M, Ludovico O, Menzaghi C, Di Paola R, Zelante L, Marucci A, et al. Low prevalence of HNF1A mutations after molecular screening of multiple MODY genes in 58 Italian families recruited in the pediatric or adult diabetes clinic from a single Italian hospital. Diabetes Care. 2014;37(12):e258-60. doi: 10.2337/dc14-1788.

95. Christian F, Angela E, Hans-Juergen W, Ulrike G-M. Renal Impairment Has No Clinically Relevant Effect on the Long-Term Exposure of Linagliptin in Patients With Type 2 Diabetes. American journal of therapeutics. 2013;20(6):618-21. doi: 10.1097/MJT.0b013e31826232dc.

96. Karges B, Bergmann C, Scholl K, Heinze E, Rasche FM, Zerres K, et al. Digenic inheritance of hepatocyte nuclear factor-1alpha and -1beta with maturity-onset diabetes of the young, polycystic thyroid, and urogenital malformations. Diabetes Care. 2007;30(6):1613-4. doi: 10.2337/dc06-2618.

97. Iwabuchi A, Kamoda T, Shinohara H, Sumazaki R. Japanese boy with maturity‐onset diabetes of the young type 3 who developed diabetes at 19 months old. Pediatrics International. 2013;55(2):e32-e4. doi: 10.1111/j.1442-200X.2012.03741.x.

98. Irgens HU, Molnes J, Johansson BB, Ringdal M, Skrivarhaug T, Undlien DE, et al. Prevalence of monogenic diabetes in the population-based Norwegian Childhood Diabetes Registry. Diabetologia. 2013;56(7):1512-9. doi: 10.1007/s00125-013-2916-y.

99. Buchbinder S, Zorn M, Bierhaus A, Nawroth PP, Müller M, Schilling T. Maturity-Onset Diabetes of the Young (MODY) caused by a novel nonsense mutation E41X in the HNF-1α gene. Exp Clin Endocrinol Diabetes. 2011;119(3):182-5. doi: 10.1055/s-0030-1262816.

100. Bazalová Z, Rypáčková B, Brož J, Brunerová L, Polák J, Rušavý Z, et al. Three novel mutations in MODY and its phenotype in three different Czech families. Diabetes Research and Clinical Practice. 2010;88(2):132-8. doi: 10.1016/j.diabres.2010.01.005.

101. Barrio R, Bellanné-Chantelot C, Moreno JC, Morel V, Calle H, Alonso M, et al. Nine novel mutations in maturity-onset diabetes of the young (MODY) candidate genes in 22 Spanish families. J Clin Endocrinol Metab. 2002;87(6):2532-9. doi: 10.1210/jcem.87.6.8530.

102. Alkorta-Aranburu G, Carmody D, Cheng YW, Nelakuditi V, Ma L, Dickens JT, et al. Phenotypic heterogeneity in monogenic diabetes: The clinical and diagnostic utility of a gene panel-based next-generation sequencing approach. Molecular genetics and metabolism. 2014;113(4):315-20. doi: 10.1016/j.ymgme.2014.09.007.

103. Kooptiwut S, Sujjitjoon J, Plengvidhya N, Boonyasrisawat W, Chongjaroen N, Jungtrakoon P, et al. Functional defect of truncated hepatocyte nuclear factor-1alpha (G554fsX556) associated with maturity-onset diabetes of the young. Biochem Biophys Res Commun. 2009;383(1):68-72. doi: 10.1016/j.bbrc.2009.03.130.

104. B. JB, U. IH, Janne M, Paweł S, Ingvild A, B. JP, et al. Targeted next-generation sequencing reveals MODY in up to 6.5% of antibody-negative diabetes cases listed in the Norwegian Childhood Diabetes Registry. Diabetologia. 2016;60(4):625-35. doi: 10.1007/s00125-016-4167-1.

105. Cappelli A, Tumini S, Consoli A, Carinci S, Piersanti C, Ruggiero G, et al. Novel mutations in GCK and HNF1A genes in Italian families with MODY phenotype. Diabetes Res Clin Pract. 2009;83(3):e72-4. doi: 10.1016/j.diabres.2008.12.007.

106. Costa A, Bescos M, Velho G, Chevre J, Vidal J, Sesmilo G, et al. Genetic and clinical characterisation of maturity-onset diabetes of the young in Spanish families. European Journal of Endocrinology. 2000;142(4):380-6. doi: 10.1530/eje.0.1420380.

107. Rozenkova K, Malikova J, Nessa A, Dusatkova L, Bjørkhaug L, Obermannova B, et al. High Incidence of Heterozygous ABCC8 and HNF1A Mutations in Czech Patients With Congenital Hyperinsulinism. J Clin Endocrinol Metab. 2015;100(12):E1540-9. doi: 10.1210/jc.2015-2763.

108. Lehto M, Wipemo C, Ivarsson SA, Lindgren C, Lipsanen-Nyman M, Weng J, et al. High frequency of mutations in MODY and mitochondrial genes in Scandinavian patients with familial early-onset diabetes. Diabetologia. 1999;42(9):1131-7. doi: 10.1007/s001250051281.

109. Bjørkhaug L, Sagen JV, Thorsby P, Søvik O, Molven A, Njølstad PR. Hepatocyte nuclear factor-1 alpha gene mutations and diabetes in Norway. J Clin Endocrinol Metab. 2003;88(2):920-31. doi: 10.1210/jc.2002-020945.
